# Effects of water quality, sanitation, handwashing, and nutritional interventions on child development in rural Kenya (WASH Benefits Kenya): a cluster-randomised controlled trial

**DOI:** 10.1016/S2352-4642(18)30025-7

**Published:** 2018-04

**Authors:** Christine P Stewart, Patricia Kariger, Lia Fernald, Amy J Pickering, Charles D Arnold, Benjamin F Arnold, Alan E Hubbard, Holly N Dentz, Audrie Lin, Theodora J Meerkerk, Erin Milner, Jenna Swarthout, John M Colford, Clair Null

**Affiliations:** a Department of Nutrition, University of California, Davis, CA, USA; b Community Health Sciences, University of California, Davis, CA, USA; c Division of Epidemiology, University of California, Davis, CA, USA; d Division of Biostatistics, University of California, Davis, CA, USA; e Environmental Health Sciences, University of California, Davis, CA, USA; f School of Public Health, University of California, Davis, CA, USA; g Department of Civil and Environmental Engineering, Stanford University, Stanford, CA, USA; h Department of Civil and Environmental Engineering, Tufts University, Medford, MA, USA; i Department of Nutrition and Health, Wageningen University, Wageningen, Netherlands; j Innovations for Poverty Action, New Haven, CT, USA; k Center for International Policy Research and Evaluation, Mathematica Policy Research, Washington DC, USA

## Abstract

**Background:**

Poor nutrition and infectious diseases can prevent children from reaching their developmental potential. We aimed to assess the effects of improvements in water, sanitation, handwashing, and nutrition on early child development in rural Kenya.

**Methods:**

In this cluster-randomised controlled trial, we enrolled pregnant women in their second or third trimester from three counties (Kakamega, Bungoma, and Vihiga) in Kenya's western region, with an average of 12 households per cluster. Groups of nine geographically adjacent clusters were block-randomised, using a random number generator, into the six intervention groups (including monthly visits to promote target behaviours), a passive control group (no visits), or a double-sized active control group (monthly household visits to measure child mid-upper arm circumference). The six intervention groups were: chlorinated drinking water; improved sanitation; handwashing with soap; combined water, sanitation, and handwashing; improved nutrition through counselling and provision of lipid-based nutrient supplements; and combined water, sanitation, handwashing, and nutrition. Here we report on the prespecified secondary child development outcomes: gross motor milestone achievement assessed with the WHO module at year 1, and communication, gross motor, personal social, and combined scores measured by the Extended Ages and Stages Questionnaire (EASQ) at year 2. Masking of participants was not possible, but data assessors were masked. Analyses were by intention to treat. This trial is registered with ClinicalTrials.gov, number NCT01704105.

**Findings:**

Between Nov 27, 2012, and May 21, 2014, 8246 women residing in 702 clusters were enrolled. No clusters were lost to follow-up, but 2212 households with 2279 children were lost to follow-up by year 2. 5791 (69%) children were measured at year 1 and 6107 (73%) at year 2. At year 1, compared with the active control group, the combined water, sanitation, handwashing, and nutrition group had greater rates of attaining the standing with assistance milestone (hazard ratio 1·23, 95% CI 1·09–1·40) and the walking with assistance milestone (1·32, 1·17–1·50), and the handwashing group had a greater rate of attaining the standing alone milestone (1·15, 1·01–1·31). There were no differences when comparing the other intervention groups with the active control group on any of the motor milestone measures at year 1. At year 2, there were no differences among groups for the communication, gross motor, personal social, or combined EASQ scores.

**Interpretation:**

The handwashing and combined water, sanitation, handwashing, and nutrition interventions might have improved child motor development after 1 year, although after 2 years there were no other differences between groups. Future research should examine ways to make community health and nutrition programmes more effective at supporting child development.

**Funding:**

Bill & Melinda Gates Foundation.

## Introduction

From gestation through age 3 years, the brain undergoes rapid growth and differentiation. Several developmental processes are sensitive to a child's early environmental inputs, including: neuronal proliferation; synapse formation, pruning, and function; myelination; axon and dendrite growth; and neuronal apoptosis.^1^ The brain architecture built during these years creates the foundation for future development and learning. However, worldwide, nearly 250 million children younger than 5 years in low-income and middle-income countries are at risk of not meeting their developmental potential due to poverty and stunted growth.^2^ Children raised in poverty are at particular risk of developmental delays because of the cumulative effects of poor nutrition, repeated and chronic infectious disease, inadequate caregiver capacities and time to invest in childcare, and psychosocial stressors within the home. Early life adversities can have long-term consequences for the developing brain, and thus can have effects on cognitive and socioemotional abilities.^2^

**Research in context**

**Evidence before this study**

At the time this study was conceptualised, there was strong evidence that risk factors such as poverty, stunting, micronutrient deficiencies, chronic or repeated infections, and inadequate care practices were associated with poor child development. However, there was limited evidence of health or nutritional interventions that improve developmental outcomes. Although we did not do a systematic review of the scientific literature before starting our trial, two reviews by Walker et al and Engle et al in *The Lancet* Series on Child Development in 2011 informed the evidence base at the time. Direct iron or iodine supplementation to micronutrient-deficient populations showed some evidence of efficacy, and a trial of lipid-based nutrient supplements in Ghana reported significant improvements in motor milestone achievement. While this study was underway, additional trials of lipid-based nutrient supplements were published suggesting positive benefits on early child development in Burkina Faso (Prado et al, 2016), Ghana (Prado et al, 2016), and Bangladesh (Matias et al, 2017), but not Malawi (Prado et al, 2016). Despite some evidence of an association between enteric infections and later cognition, there was no published evidence that water, sanitation, or hygiene interventions that might prevent enteric infections improve early child development outcomes. Since this study began, one intensive handwashing promotion trial in Pakistan (Bowen et al, 2012) provided evidence of significant developmental benefits 6 years later when children were aged 6–7·5 years.

**Added value of this study**

This trial provides some of the first evidence from a randomised controlled trial that an integrated water, sanitation, handwashing, and nutrition intervention improved motor development in children at 1 year of age but did not improve measures of child development assessed at 2 years of age.

**Implications of all the available evidence**

This study provided evidence of only a modest effect on gross motor development outcomes at 1 year from baseline. However, measures of intervention adherence were variable (high for nutrition and sanitation interventions, but low for other interventions). As compared with other trials that had had a high intensity of contact between caregivers and health promoters, this trial had a lower frequency of contact (ie, monthly), which might be more similar to what government or donor-funded programmes might be able to achieve at scale. This lower intensity might have limited the potential for the interventions to improve child development. Frequent contact with health promoters might lead to higher adherence to the recommended behaviours. It could also provide added benefits with respect to social support for the caregiver or encouraging greater attention to be paid to the child if combined with the promotion of responsive stimulation, which might contribute to improved developmental outcomes.

There is a large and growing body of evidence linking inadequate nutrition in early childhood to impaired concurrent development and long-term functioning. Stunting in children younger than 2 years has been associated with lower developmental scores in early childhood, poorer cognition and executive function in middle childhood and adolescence, fewer years of school completion, and lower income in adulthood.^2^ Deficiencies in micronutrients, especially iron and iodine, are also associated with worse outcomes throughout the lifecourse, and might be irreversible, even with treatment.^1^, ^3^, ^4^, ^5^ However, early care practices or interventions can prevent or reduce the effect of nutritional risks on development. Breastfeeding has been associated with greater intelligence quotient scores measured in childhood and adolescence,^6^ and has been shown to be a protective factor for children exposed to one or more developmental risks.^5^ Trials of nutritional interventions, including micronutrient supplementation during pregnancy or early childhood and provision of micronutrient-fortified complementary foods or lipid-based nutrient supplements, have also shown some benefits on developmental outcomes in children.^7^

The literature linking water, sanitation, or handwashing interventions with developmental outcomes is sparse. One study of an intensive handwashing promotion intervention in Pakistan noted significant developmental benefits in 5–7-year-old children whose families had participated in the trial when their children were younger than 30 months.^8^ Despite the limited evidence from trials, there are plausible biological pathways through which water, sanitation, or handwashing interventions might affect child development by reducing infection and inflammation in pregnancy or early childhood.^9^ Frequent diarrhoeal illness is associated with growth faltering,^10^ which could lead to developmental impairments. Two longitudinal studies presented evidence of an association between *Cryptosporidium* or *Giardia* infection and diarrhoea in early life with cognitive function in later life.^11^, ^12^ Frequent febrile illness or elevated inflammatory biomarkers have also been associated with poor cognitive, language, and motor development.^13^ Chronic enteropathogen exposure might lead to sustained inflammation, which could activate microglia and perivascular macrophages, leading to a neuroinflammatory state.^14^ Inflammation will also cause a decrease in plasma iron concentrations because of an inhibition of iron absorption and sequestration of iron in splenic and hepatic macrophages,^9^ thereby limiting its availability for normal developmental processes that rely on iron. Finally, frequent days of illness might inhibit child exploration and positive interactions with caregivers, hindering opportunities for engagement in stimulating activities that promote development.

The WASH Benefits trial in Kenya was designed to assess the independent and combined effects of water, sanitation, handwashing, and nutritional interventions on child growth, health, and development after 2 years of intervention. We recently reported small improvements in growth with improved nutrition, but no effect of study interventions on diarrhoea, in the same trial setting.^15^ A companion trial in Bangladesh has reported significant effects on growth with improved nutrition, reductions in diarrhoea with improvements in water, sanitation, handwashing, or nutrition,^16^ and improvements in indicators of child development across all intervention groups.^17^ The objective of the present analysis was to evaluate two hypotheses. First, interventions improving water quality; sanitation; handwashing with soap; water, sanitation, and handwashing in combination; nutrition; or water, sanitation, handwashing, and nutrition in combination would improve indicators of child development during the first 2 years of life. Second, the combination of water, sanitation, handwashing, and nutrition would improve child development measurements more than combined water, sanitation, and handwashing, or more than nutrition alone.

## Methods

### Study design

Details on the study methods have been published previously.^15^, ^18^ The Kenya WASH Benefits study was a cluster-randomised trial done in three counties (Kakamega, Bungoma, and Vihiga) in Kenya's western region. Village clusters comprising an average of 12 enrolled households each were randomly assigned by geographical blocks into one of eight study groups: chlorine treatment of drinking water; improved sanitation limiting exposure to faeces; handwashing with soap; combined water, sanitation, and handwashing interventions; infant and young child feeding counselling plus small-quantity lipid-based nutrient supplements (nutrition group); combined water, sanitation, handwashing, and nutrition; active control; and passive control. In all groups except for the passive control, community health promoters were instructed to do monthly home visits to measure child mid-upper arm circumference. In the active control group, this was the only activity, whereas in the other intervention groups, community health promoters used additional behaviour change techniques to target the group-specific behaviours. The passive control group was included to distinguish the effects of interaction with the community health promoters from the effects of the water, sanitation, handwashing, and nutrition components of the interventions.

The trial protocol was approved by human subjects' committees at the University of California, Berkeley, CA, USA, Stanford University, CA, USA, and the Kenya Medical Research Institute, Nairobi, Kenya.

### Participants

Study eligibility was determined at the community and individual levels. Villages were eligible for selection into the study if they were rural, most of the population relied on communal water sources and had unimproved sanitation facilities, and there were no other ongoing combined water, sanitation, and handwashing or nutrition programmes. Households were eligible for participation if there was a woman in her second or third trimester of pregnancy who planned to reside in the community for at least 2 years and who could speak Kiswahili, Luhya, or English. A minimum of six eligible pregnant women was required to form a study cluster, which could contain up to three neighbouring villages. Mothers provided written informed consent for themselves and their infants.

### Randomisation and masking

Clusters were randomly assigned to intervention groups at the University of California, Berkeley, CA, USA, using a random number generator. Groups of nine geographically adjacent clusters were block-randomised into the six intervention groups, passive control group, or double-sized active control group. Participants and other community members were informed of their intervention group assignment after the baseline survey. Masking of participants was not possible in view of the nature of the interventions. Data collectors who assessed the study outcomes were not informed of the cluster intervention assignment, but might have inferred the assignment.

### Procedures

Community health promoters were nominated by mothers in the community and trained to provide intervention group-specific behaviour change activities as well as instructions on hardware use or provision of supplements. They were also trained to measure child mid-upper arm circumference to identify and provide referrals for potential cases of severe acute malnutrition, as well as to increase familial involvement and interest in care practices concerning their child's growth and health. Training varied in length, ranging from 3 days for the active control group to 7 days for the combined intervention groups. Group-specific refresher trainings were done every 6 months thereafter. Supportive supervision was provided through in-person, one-on-one spot-checks by supervisors, and via phone and text message support. Health promoters were provided a nominal stipend for their activities of approximately US$15 per month for the first 6 months when they had more intensive engagement with the study participants, and $9 per month thereafter (the prevailing daily wage for unskilled labour in the study area is $1–2). A second promoter was recruited in larger clusters (more than ten for single groups and more than eight for combined groups).

Each intervention consisted of a comprehensive behaviour change package of key messages; visual aids in the form of flipcharts, posters, and reminder cue cards; interactive activities with songs, games, or pledges to commit to practice target behaviours; and the distribution of group-specific hardware, products, or supplements. Intervention-specific promoter training materials, visit plans, and visual aids are available online. Promoters were encouraged to visit at least once a month throughout the duration of the 2 year trial. Visit modules varied in length but were designed to last less than 1 h.

In the three intervention groups including water quality improvements, community health promoters advocated drinking water treatment with sodium hypochlorite using either chlorine dispensers installed at the point of collection in study villages or bottled chlorine provided directly to households. Community health promoters used chlorine test strips to spot-check household chlorine concentrations during monthly visits and results were used to improve counselling.

In the three intervention groups including sanitation, existing latrines were upgraded and improved by installing a plastic slab with a tight-fitting lid; households without latrines or with poor quality latrines were provided with a new latrine. All households in study compounds were provided with a plastic potty for each child younger than 3 years as well as a sani-scoop with a paddle to remove animal and human faecal material from the yard surrounding the home.

In the three intervention groups including handwashing, households were provided with two handwashing stations, one near the latrine and a second near the cooking area. Stations were constructed with two foot-pedal operated jerry cans that could be tipped to dispense a small stream of either soapy water or rinse water. Households were responsible for keeping the tanks stocked with water, but the community health promoters refilled soap roughly every 3 months.

In the two intervention groups including nutrition, micronutrient-fortified, small quantity lipid-based nutrient supplements were provided to children aged 6–24 months. The appendix provides nutrient composition of the supplements. Community health promoters delivered monthly rations of lipid-based nutrient supplements in the form of 10 g sachets and caregivers were instructed to mix the contents of the sachet into the child's complementary foods twice a day. Key messages for maternal, infant, and young child feeding consisted of dietary diversity during pregnancy and lactation, early initiation of breastfeeding, exclusive breastfeeding from 0–6 months and continued breastfeeding through 24 months, timely introduction of complementary foods, dietary diversity, feeding frequency, and feeding during illness. Midway through the trial, a decision was made to distribute the lipid-based nutrient supplements by higher level project staff to improve the quality of data recording on distributions and adherence.

After consent and enrolment, a baseline survey was administered with questions about household socioeconomic status; animal ownership; water, sanitation, and hygiene behaviours and conditions within the home; household food insecurity using the Household Hunger Scale; and household size and demographics. At the baseline, year 1, and year 2 surveys, data were collected on access and use of the interventions, including health promoter visits, chlorine treatment of drinking water, access to an improved latrine, child faeces disposal, presence of soap and water at the handwashing stations, and consumption rate of lipid-based nutrient supplements. Access to improved sources of drinking water or sanitation was defined using the WHO/UNICEF Joint Monitoring Program categorisations.

### Outcomes

Here we report on the child development outcomes, prespecified secondary outcomes of the trial.^18^ Trained enumerators did follow-up visits at two timepoints—1 and 2 years after the start of intervention activities in study communities. At 1 year, child gross motor milestone achievement was assessed using the WHO module.^19^ The questions asked about six behaviours: sitting without support; standing with assistance; hands-and-knees crawling; walking with assistance; standing alone; and walking alone. Interviewers described each milestone and showed pictures to caregivers, who were then asked to report if the child was able to do the behaviour and if so, whether they were recorded doing the behaviour in the past 24 h. The report of ever performing the behaviour was used in the analysis. Enumerators were trained over a 2 day period and standardised using video recordings.

To assess communication, gross motor, and personal social development at year 2, we used the corresponding subscales of the Extended Ages and Stages Questionnaire (EASQ), which is a tool that an investigator on our team (PK) adapted from Squires and Bricker^20^ for use in low-income and middle-income country contexts.^21^ The adapted method is a parental report measure of child development, which also includes opportunities for the child to demonstrate behaviours and skills. Specific demonstration items are described in the appendix. The communication subscale assesses language development and verbal abilities; the gross motor subscale assesses skills such as walking, jumping, and kicking; and the personal social subscale assesses emotional and behavioural capacities. The fine motor and problem solving subscales of the EASQ were not included in the test adaptation because of the difficulty in administering them in large-scale surveys. For all items, respondents could reply with one of three responses: yes, sometimes, or not yet. The EASQ was translated, piloted, and adapted according to recommended procedures.^22^ Enumerators were trained and standardised during an 8 day training course. Every enumerator completed two or three inter-observer administrations in which their responses were compared with an identified gold standard. The average proportion agreement ranged from 0·92–0·99. To create the reference distributions for each of the EASQ communication, gross motor, and personal social sub-scales and the overall combined scale, the summed age-specific raw scores from the double-sized active control group were standardised to have mean 0 and standard deviation 1, yielding *Z* scores for each 2 month age band. *Z* scores for the children who were not in the active control group were created using the relevant reference distribution for each age band. For both the motor milestone and EASQ assessments, children with disability in hearing, seeing, motor function, or other physical disabilities were excluded.

### Statistical analysis

The rationale for the sample size in the main trial is described in detail elsewhere.^18^ In brief, the sample size was chosen to detect a difference of 0·15 in the primary outcome of length-for-age *Z* score assuming a type 1 error (α) of 0·05, power (1–β) of 0·8, and a 10% loss to follow-up after baseline. This design had more than 80% power to detect a difference in EASQ *Z* scores of 0·15 for any single comparison (80 clusters) against the double-sized control (160 clusters), assuming nine children per cluster, a two-sided α of 0·05, and intracluster correlation of 0·05 using a standard equation for cluster randomised trials.^23^ Under the same assumptions, the minimum detectable effect for comparison of combined versus single intervention groups for the EASQ *Z* scores was 0·17.

We compared acquisition rates for each of the six WHO motor milestones. We modelled milestone acquisition rates using a semiparametric Cox-proportional hazards model, which was estimated from current status data using a generalised additive model with complementary log–log link and baseline hazard fit with a monotonic cubic spline.^24^, ^25^

For all analyses, we considered the intention-to-treat, unadjusted differences between each intervention group and the active control, and differences between the combined water, sanitation, handwashing, and nutrition group versus the nutrition group or versus the combined water, sanitation, and handwashing group as our primary inference. For analysis of EASQ scores, we used targeted maximum likelihood estimation to model the mean difference between each intervention group and the active control group, and used influence curve-based standard errors that accounted for the geographically matched, cluster-randomised design.^26^ The targeted maximum likelihood approach enabled us to flexibly adjust for prespecified covariates to potentially gain precision in the adjusted analysis, and to account for potentially informative censoring due to attrition (additional details in analysis plan are available online). All analyses were prespecified, and two statisticians (CDA and JS) separately analysed the data and compared results. Both statisticians remained masked to intervention group assignment until all analyses and results had been replicated. Analyses were done with Stata (version 14.1) and R (version 3.3.2).

The trial is registered at ClinicalTrials.gov, number NCT01704105.

### Role of the funding source

The funder reviewed and approved the study design, but had no role in data collection, data analysis, data interpretation, or writing of the report. The corresponding author had full access to all of the data in the study and had final responsibility for the decision to submit for publication.

## Results

**Figure 1:**
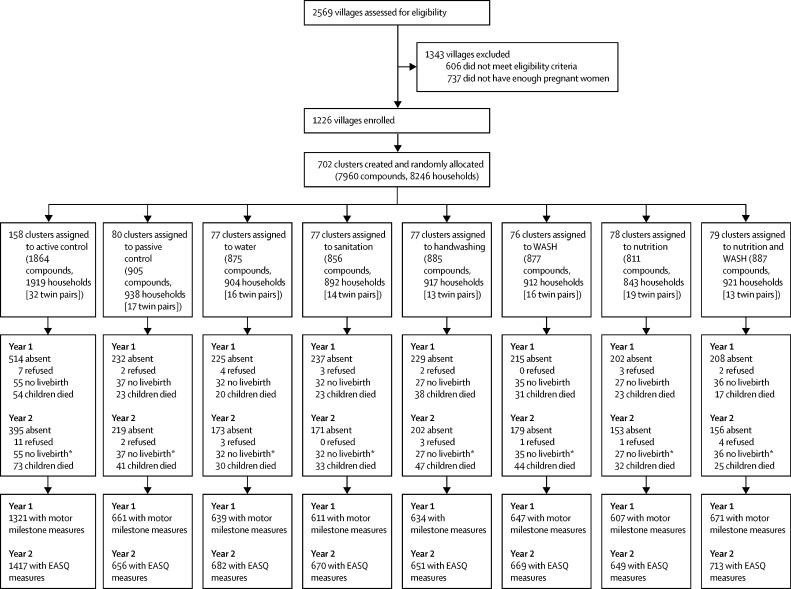
Trial profile Numbers are children except where specified. Attrition was at the individual level. WASH=water, sanitation, and handwashing. EASQ=Extended Ages and Stages Questionnaire. *Births and deaths reported in year 2 in the figure are cumulative.

Between Nov 27, 2012, and May 21, 2014, 8246 households residing in 702 clusters were enrolled in the study. 281 pregnant women did not have a livebirth and 140 delivered twins. No clusters were lost to follow-up, but 2212 households with 2279 children were lost to follow-up by year 2. 5791 (70%) children were measured at year 1 and 6107 (74%) at year 2. Losses to follow-up were balanced across groups (figure 1).

Household characteristics at enrolment were similar across groups (table 1). On average, mothers were 26 years old and nearly half had completed at least primary school education. A higher proportion of fathers (about 60%) had completed the same level of schooling. Most households had access to an improved water source and nearly all adults reported using a latrine for defecation. Open defecation was more common in children than adults. Reported household hunger was less common, with only about 10% reporting moderate to severe hunger.

**Table 1:** Baseline characteristics

		**Active control group (N=1919)**	**Passive control group (N=938)**	**Water group (N=904)**	**Sanitation group (N=892)**	**Handwashing group (N=917)**	**Water, sanitation, and handwashing group (N=912)**	**Nutrition group (N=843)**	**Water, sanitation, handwashing, and nutrition group (N=921)**
**Maternal**									
Age (years)		26 (6)	26 (7)	26 (6)	26 (7)	26 (6)	26 (6)	26 (6)	26 (6)
Height (cm)		160 (6)	160 (6)	160 (6)	160 (6)	160 (6)	160 (6)	160 (6)	160 (6)
Study child is firstborn		490 (26%)	237 (25%)	205 (23%)	222 (25%)	208 (23%)	191 (21%)	206 (24%)	225 (25%)
Completed at least primary education		916 (48%)	441 (47%)	447 (49%)	430 (48%)	402 (44%)	430 (47%)	409 (49%)	438 (48%)
**Paternal**									
Completed at least primary education		1098 (62%)	521 (60%)	532 (64%)	482 (58%)	500 (59%)	521 (61%)	491 (64%)	526 (62%)
Works in agriculture		749 (41%)	376 (43%)	378 (44%)	362 (43%)	363 (42%)	374 (43%)	343 (43%)	372 (43%)
**Household**									
Number of people per compound		8 (5)	8 (6)	8 (6)	8 (5)	8 (6)	8 (5)	8 (7)	8 (5)
Number of children aged <18 years		3 (2)	3 (2)	3 (2)	3 (2)	3 (2)	3 (2)	3 (2)	3 (2)
Has electricity		122 (6%)	51 (5%)	60 (7%)	73 (8%)	67 (7%)	64 (7%)	58 (7%)	67 (7%)
Has a cement floor		107 (6%)	50 (5%)	73 (8%)	49 (5%)	41 (4%)	50 (5%)	48 (6%)	56 (6%)
Has an iron roof		1302 (68%)	600 (64%)	610 (67%)	587 (66%)	581 (63%)	574 (63%)	581 (69%)	615 (67%)
**Drinking water**									
One-way walking time to primary water source (min)		11 (12)	12 (16)	12 (30)	10 (10)	11 (13)	11 (13)	11 (12)	11 (12)
Primary drinking water source is improved*		1446 (76%)	699 (75%)	679 (75%)	675 (76%)	708 (78%)	624 (69%)	603 (72%)	697 (76%)
Reported treating currently stored water		196/1557 (13%)	92/747 (12%)	81/732 (11%)	94/728 (13%)	96/757 (13%)	97/724 (13%)	79/682 (12%)	106/743 (14%)
**Sanitation**									
Usually defecate in primary toilet		1767 (94%)	867 (95%)	825 (94%)	806 (94%)	839 (94%)	850 (95%)	780 (95%)	849 (94%)
Daily defecating in the open, children aged 0 to <3 years		789/1017 (78%)	378/492 (77%)	376/469 (80%)	370/493 (75%)	358/469 (76%)	394/515 (77%)	363/462 (79%)	388/497 (78%)
Latrine									
	Own any latrine	1561 (82%)	774 (83%)	750 (83%)	722 (81%)	756 (83%)	754 (83%)	701 (83%)	764 (83%)
	Access to improved latrine	309 (17%)	153 (17%)	150 (18%)	131 (16%)	157 (19%)	153 (18%)	119 (15%)	143 (16%)
Human faeces observed in compound		163 (9%)	79 (8%)	66 (7%)	72 (8%)	84 (9%)	73 (8%)	73 (9%)	87 (9%)
**Handwashing location**									
Has water within 2 m		487 (25%)	236 (25%)	242 (27%)	245 (28%)	245 (27%)	251 (28%)	228 (27%)	249 (27%)
Has soap within 2 m		164 (9%)	94 (10%)	91 (10%)	75 (8%)	83 (9%)	115 (13%)	90 (11%)	87 (9%)
**Food security**									
Moderate to severe household hunger†		203 (11%)	113 (12%)	106 (12%)	91 (10%)	92 (10%)	101 (11%)	98 (12%)	104 (11%)

Data are n (%) or mean (SD). Missing <8% of observations unless denominator indicated.

* Defined by WHO/UNICEF Joint Monitoring Program's definition for an improved water source.

† Assessed by the Household Hunger Scale. Slight discrepancies between data in this table and those presented in Null, Stewart, and Pickering et al, 2018, are due to data cleaning that occurred between the time of the analyses.

2788 (74%) of 3769 households had been visited by a community health promoter in the past month during the first year, which dropped to 2004 (37%) of 5456 in the second year (appendix). These proportions did not differ by intervention group and were also similar to the active control group. In the nutrition groups, parental report of lipid-based nutrient supplement adherence was high at both year 1 and year 2, with 10 847 (95%) of 11 396 and 7605 (115%) of 6594, respectively, of the expected sachets reported to have been consumed in the past week (measured as the reported number of sachets consumed in the past week divided by the expected number of 14 sachets). Objective indicators of intervention compliance in the intervention groups including water, sanitation, and handwashing were variable over time. In the water groups, 487 (41%) of 1176 households had detectable chlorine in stored drinking water at year 1, dropping to 384 (20%) of 1887 at year 2. Access to an improved latrine was high, in roughly 80% of households at year 1 and year 2 in the sanitation groups (1306 [89%] of 1461 and 1624 [80%] of 2033, respectively). Soap was observed in a handwashing station at 1178 (77%) of 1530 households in year 1 and 441 (21%) of 2100 households at year 2 in the handwashing groups.

**Figure 2:**
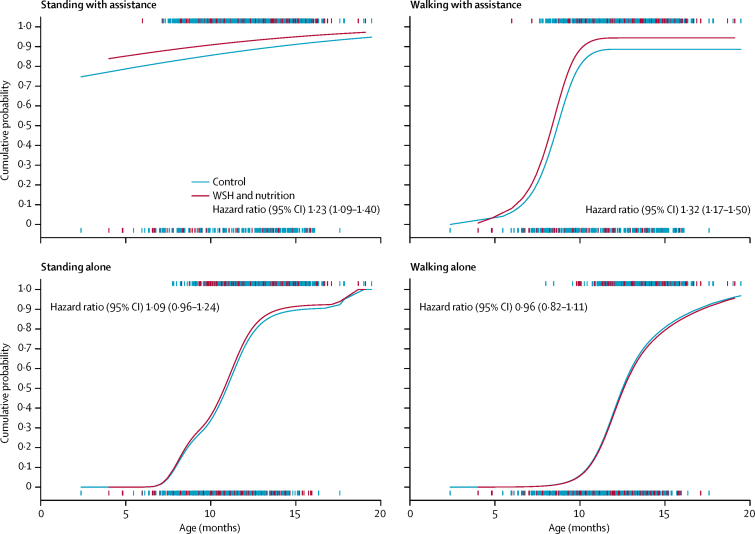
Cumulative probability of children who achieved four of the developmental milestones after 1 year of intervention in the WASH and nutrition group compared with the active control group Hash marks indicate the age of observed children who had achieved the milestone (1·0) or not achieved the milestone (0·0). WASH=water, sanitation, and handwashing.

At the year 1 follow-up, the median age of children was 1·0 year (IQR 0·9–1·2). The age of attainment of each of the motor milestones in the study group was similar to the WHO reference population (table 2). Because more than 95% of children had achieved the sitting with assistance and the hands-and-knees crawling milestones by the 1 year follow-up, we did not make statistical comparisons between groups for these outcomes. Compared with the active control group, the combined water, sanitation, handwashing, and nutrition group had greater rates of milestone achievement for standing with assistance (hazard ratio [HR] 1·23, 95% CI 1·09–1·40) and walking with assistance (1·32, 1·17–1·50; figure 2), and the handwashing group had a greater rate of attainment for the standing alone milestone (HR 1·15, 95% CI 1·01–1·31). No other differences were evident (table 3). Estimates did not change appreciably after adjustment for covariates (appendix).

**Table 2:** Estimated age of attainment for each of the motor milestones among children in the study sample compared with the WHO reference population

	WHO growth standards reference*, **median (IQR) age of attainment, months**	**WASH Benefits study children**†, **median (IQR) age of attainment, months**	**Age 6–8 months (N=315)**	**Age 9–11 months (N=2054)**	**Age 12–14 months (N=2878)**	**Age 15–17 months (N=505)**
Sitting without support	5·9 (5·8–6·0)	··	313 (99%)	2048 (100%)	2863 (99%)	503 (100%)
Standing with assistance	7·4 (6·6–8·4)	7·4 (6·5–8·4)	211 (67%)	1885 (92%)	2604 (90%)	444 (88%)
Hands-and-knees crawling	8·3 (8·2–8·4)	··	252 (80%)	1982 (97%)	2871 (97%)	482 (95%)
Walking with assistance	9·0 (8·2–10·0)	8·6 (7·5–9·7)	122 (39%)	1743 (85%)	2584 (90%)	441 (87%)
Standing alone	10·8 (9·7–12·0)	10·7 (9·4–12·1)	31 (10%)	1057 (52%)	2426 (84%)	475 (94%)
Walking alone	12·0 (11·0–13·0)	12·5 (11·4–14·3)	5 (2%)	371 (18%)	1812 (63%)	423 (84%)

* Published data from the WHO Multicentre Growth Reference Study.^19^

† Estimated from modelled cumulative probability of attainment. Median age could not be estimated for the sitting without support and hands-and-knees crawling milestones because >95% of children had already achieved this milestone prior to the assessment.

**Table 3:** Relative rate of motor milestone attainment in each of the intervention groups

	**N**	**Hazard ratio *vs* active control group (95% CI)**	**Hazard ratio *vs* WASH group (95% CI)**	**Hazard ratio *vs* nutrition group (95% CI)**
**Standing with assistance**				
Active control	1321	1 (ref)	··	··
Passive control	661	0·98 (0·87–1·10)	··	··
Water	639	1·05 (0·93–1·18)	··	··
Sanitation	611	0·99 (0·88–1·12)	··	··
Handwashing	634	1·06 (0·93–1·19)	··	··
WASH	647	1·06 (0·94–1·20)	1 (ref)	··
Nutrition	607	1·07 (0·94–1·21)	··	1 (ref)
WASH and nutrition	671	1·23 (1·09–1·40)	1·17 (1·01–1·35)	1·16 (1·00–1·35)
**Walking with assistance**				
Active control	1321	1 (ref)	··	··
Passive control	661	0·96 (0·85–1·09)	··	··
Water	639	0·95 (0·84–1·07)	··	··
Sanitation	611	1·01 (0·89–1·15)	··	··
Handwashing	634	1·07 (0·94–1·21)	··	··
WASH	647	1·00 (0·89–1·13)	1 (ref)	··
Nutrition	607	1·12 (0·99–1·27)	··	1 (ref)
WASH and nutrition	671	1·32 (1·17–1·50)	1·32 (1·14–1·53)	1·18 (1·02–1·37)
**Standing alone**				
Active control	1321	1 (ref)	··	··
Passive control	661	1·12 (0·98–1·27)	··	··
Water	639	0·98 (0·86–1·11)	··	··
Sanitation	611	1·04 (0·91–1·18)	··	··
Handwashing	634	1·15 (1·01–1·31)	··	··
WASH	647	1·07 (0·94–1·22)	1 (ref)	··
Nutrition	607	1·08 (0·94–1·23)	··	1 (ref)
Water, sanitation, handwashing, and nutrition	671	1·09 (0·96–1·24)	1·02 (0·88–1·18)	1·02 (0·88–1·19)
**Walking alone**				
Active control	1321	1 (ref)	··	··
Passive control	661	1·06 (0·91–1·22)	··	··
Water	639	0·95 (0·82–1·10)	··	··
Sanitation	611	0·93 (0·80–1·09)	··	··
Handwashing	634	1·06 (0·92–1·23)	··	··
WASH	647	1·04 (0·90–1·21)	1 (ref)	··
Nutrition	607	1·03 (0·89–1·20)	··	1 (ref)
WASH and nutrition	671	0·96 (0·82–1·11)	0·92 (0·78–1·10)	0·93 (0·78–1·11)

WASH=water, sanitation, and handwashing. Ref=reference.

At the year 2 follow-up, the median age for children was 2·1 years (IQR 1·9–2·2). Intracluster correlation coefficients for the EASQ scores were as follows: communication 0·078; gross motor 0·119; personal social 0·080; and combined score 0·124. There were no apparent differences between groups for the communication, gross motor, personal social, or combined EASQ scores, with one exception (table 4). The sanitation group had a significantly lower gross motor score (−0·10 SD, 95% CI −0·19 to 0·00) and a lower combined score (−0·11 SD, 95% CI −0·21 to −0·01) compared with the active control group. However, after adjustment for covariates, this difference was attenuated and no longer significant (appendix). There were no other substantive changes in the effect size estimates after adjustment for baseline covariates or in inverse probability of censoring weighted analysis (appendix).

**Table 4:** Standardised differences in scores on the communication, gross motor, personal social, and combined scales of the Extended Ages and Stages Questionnaire

	**N, mean (SD)**	**Mean difference *vs* active control group (95% CI)**	**Mean difference *vs* WASH group (95% CI)**	**Mean difference *vs* nutrition group (95% CI)**
**Communication *Z* score**				
Active control	1417, 0·00 (1·00)	1 (ref)	··	··
Passive control	656, −0·05 (0·99)	−0·05 (−0·15 to 0·05)	··	··
Water	682, −0·01 (1·01)	0·00 (−0·11 to 0·11)	··	··
Sanitation	670, −0·08 (0·99)	−0·08 (−0·17 to 0·02)	··	··
Handwashing	651, −0·03 (0·99)	−0·04 (−0·15 to 0·07)	··	··
WASH	669, 0·02 (0·98)	0·03 (−0·08 to 0·14)	1 (ref)	··
Nutrition	649, 0·04 (0·99)	0·04 (−0·07 to 0·15)	··	1 (ref)
WASH and nutrition	713, −0·03 (0·98)	−0·02 (−0·11 to 0·08)	−0·05 (−0·17 to 0·06)	−0·04 (−0·15 to 0·06)
**Gross motor *Z* score**				
Active control	1417, 0·00 (1·00)	1 (ref)	··	··
Passive control	656, 0·00 (0·98)	0·00 (−0·11 to 0·11)	··	··
Water	682, 0·02 (0·99)	0·02 (−0·10 to 0·13)	··	··
Sanitation	670, −0·11 (1·04)	−0·10 (−0·19 to 0·00)	··	··
Handwashing	651, −0·02 (1·03)	−0·04 (−0·16 to 0·08)	··	··
WASH	669, 0·02 (0·92)	0·01 (−0·10 to 0·11)	1 (ref)	··
Nutrition	649, 0·04 (0·95)	0·02 (−0·08 to 0·13)	··	1 (ref)
WASH and nutrition	713, −0·03 (0·96)	−0·03 (−0·13 to 0·07)	−0·05 (−0·17 to 0·07)	−0·05 (−0·16 to 0·06)
**Personal social *Z* score**				
Active control	1417, 0·00 (1·00)	1 (ref)	··	··
Passive control	656, 0·01 (0·98)	0·00 (−0·10 to 0·10)	··	··
Water	682, −0·04 (1·02)	−0·03 (−0·16 to 0·09)	··	··
Sanitation	670, −0·09 (1·01)	−0·09 (−0·20 to 0·01)	··	··
Handwashing	651, −0·04 (1·02)	−0·04 (−0·15 to 0·08)	··	··
WASH	669, −0·02 (0·98)	−0·03 (−0·13 to 0·07)	1 (ref)	··
Nutrition	649, 0·02 (0·96)	0·01 (−0·10 to 0·12)	··	1 (ref)
WASH and nutrition	713, 0·02 (0·97)	0·02 (−0·08 to 0·11)	0·05 (−0·07 to 0·16)	0·01 (−0·10 to 0·12)
**Combined *Z* score**				
Active control	1417, 0·00 (1·00)	1 (ref)	··	··
Passive control	656, −0·02 (0·98)	−0·03 (−0·13 to 0·07)	··	··
Water	682, −0·01 (1·00)	−0·01 (−0·12 to 0·11)	··	··
Sanitation	670, −0·11 (1·02)	−0·11 (−0·21 to −0·01)	··	··
Handwashing	651, −0·03 (1·01)	−0·04 (−0·16 to 0·07)	··	··
WASH	669, 0·01 (0·95)	0·01 (−0·10 to 0·11)	1 (ref)	··
Nutrition	649, 0·04 (0·98)	0·03 (−0·08 to 0·15)	··	1 (ref)
WASH and nutrition	713, −0·02 (0·97)	−0·02 (−0·12 to 0·08)	−0·03 (−0·15 to 0·09)	−0·04 (−0·15 to 0·07)

WASH=water, sanitation, and handwashing.

Interaction tests among study group and child sex, maternal parity, maternal age, maternal education, household hunger score, and socioeconomic status yielded some significant tests (p<0·05), yet no consistent patterns were apparent in stratified subgroup analyses (appendix).

## Discussion

In this trial of independent and combined water, sanitation, handwashing, and nutritional interventions provided to households in rural Kenya, we found limited evidence of intervention effects on child development outcomes. After 1 year of intervention, there were small, significant improvements in prespecified motor development milestones in the combined water, sanitation, handwashing, and nutrition group on standing or walking with assistance, but this difference did not consistently carry forward into motor or other child development measures assessed 1 year later. In the handwashing group, there was a small improvement in the standing alone motor milestone measured at year 1, but no other improvements in any other outcome measure at that point or at the 2 year follow-up.

As a whole, this cohort of Kenyan children had a similar age of attainment of the motor milestones as compared with the WHO reference population.^19^ Nevertheless, we noted greater attainment rates in the combined water, sanitation, handwashing, and nutrition group. This group, together with the nutrition group, also had significantly higher length-for-age and weight-for-age *Z* scores, and correspondingly lower prevalence of stunting and underweight.^15^ Thus, it is possible that some of the observed effect may have been mediated through improved growth.

Other complementary feeding trials, particularly those of lipid-based nutrient supplements during complementary feeding, have reported mostly positive effects on developmental outcomes. A trial in Burkina Faso—in which 9-month-old children were provided with various formulations of lipid-based nutrient supplements together with morbidity monitoring and treatment of malaria and diarrhoea—reported that, at 18 months of age, children scored significantly higher on the motor, language, and personal social development domains of the Developmental Milestones Checklist II, with effect sizes of roughly 0·3 SD each.^27^ Similarly, two trials in Ghana noted improvements in motor development at 1 year of age in children who received lipid-based nutrient supplements in comparison to a non-intervention group.^28^, ^29^ In a study in which children were provided with either lipid-based nutrient supplements or multiple micronutrient powder compared with a control group, improvements in motor and receptive language were recorded in both intervention groups.^30^ Similarly, in the WASH Benefits study in Bangladesh using the EASQ assessment, there were improvements in motor, language, and personal social development in both of the nutrition groups that received lipid-based nutrient supplements.^17^ Finally, a large-scale evaluation of the Alive and Thrive Program in Bangladesh, an intensive behaviour change communication intervention promoting improved complementary feeding practices, also found significant improvements in language and gross motor development in children aged 6–48 months.^31^ In contrast to these studies finding positive effects of lipid-based nutrient supplements or nutrition education on child development, two trials in Malawi supplementing children from age 6 months to 18 months found no difference on gross motor, language, or socio-emotional development measures at 18 months of age.^32^, ^33^ The researchers speculated that the lack of benefits could have been due to differences in care practices, such as early introduction of complementary foods in Malawi that might have contributed to increased illness across all groups, obscuring any positive effect of lipid-based nutrient supplements on growth and development. Alternatively, the investigators suggested that the outcome measure might have lacked adequate sensitivity to detect developmental differences between the groups.

Our results stand in contrast to the two water, sanitation, and handwashing trials that have assessed child development outcomes. In Pakistan, a trial of intensive handwashing promotion was associated with long-term improvements in child developmental outcomes, assessed 5 years after the trial ended.^8^ The WASH Benefits trial in Bangladesh found increases of approximately 0·10–0·35 SD in motor, language, and personal social development across most of the individual and combined intervention groups.^17^

There are some important differences between our trial and the studies that have reported improved developmental outcomes. First, there was a much higher intensity of contact with the health promoters in many of the other trials. In the Bangladesh WASH Benefits trial, for example, the frequency of contacts was six visits per month throughout the trial.^16^, ^17^ In the Pakistan handwashing trial, the frequency of contacts was at least twice a week.^8^ The level of contact in Kenya was also lower than in the Burkina Faso and Ghana lipid-based nutrient supplements trials, which included weekly or bimonthly visits for lipid-based nutrient supplements distribution and morbidity surveillance and treatment.^27^, ^29^ In the Alive and Thrive programme, the frequency of contacts with infant and young child feeding promoters and community health volunteers totalled about two visits per month during the child's first year of life.^31^

A high frequency of contact with community health promoters might lead to greater uptake of or adherence to the targeted intervention behaviours. It might also provide knock-on benefits, in addition to the water, sanitation, handwashing, or nutrition-specific messages and counselling provided. It might increase support for caregiving, particularly if paired with messages on responsive parenting. Studies of positive parenting or early child stimulation programmes have generally shown positive effects on children's cognitive and language development, with effect size improvements of approximately 0·3 SD.^7^ Furthermore, developing a supportive relationship with a peer in the community might improve maternal mental health or resilience in the face of multiple stressors, as well as build confidence and self-efficacy to provide nurturing care for children.^34^

We included two control groups in the study design: an active control with community health promoter visits at a similar frequency to the intervention groups, and a passive control with no community health promoter contacts. This was done to isolate the potential effects of community health promoter contact on the measured outcomes. We recorded no differences in measures of child development in the active control relative to the passive control group. However, the infrequency of contact during implementation—only once per month in the first year and roughly once per 2 months in the second year—hinders our ability to effectively test this hypothesis.

The other studies reporting larger benefits also had higher uptake of the targeted behaviours than in our study. In the water and handwashing intervention groups, objective indicators of behaviour change suggested that most households were not practicing the target behaviours for the full 2 year follow-up. By contrast, lipid-based nutrient supplements adherence was high, and coverage of improved sanitation infrastructure and tools for safe management of children's faeces was high. The small effect on motor development and lack of effect on other developmental measures observed in this trial could be due to low uptake, limiting the potential for the child to benefit. In the WASH Benefits Bangladesh trial, uptake was higher for many of the key behaviours and there were significantly higher developmental scores across nearly all intervention groups compared with passive control.^17^ Therefore, it is possible that if there had been more sustained uptake in this study, particularly in year 2 when the EASQ assessments were administered, there might have been greater benefits.

Another possible explanation for our findings is that we recorded minimal effects of the interventions on measures on the causal pathway connecting water, sanitation, handwashing, or nutrition and child development. For example, there were no effects of the interventions on diarrhoeal disease in any groups, and only a small improvement on child growth in the nutrition groups.^15^ However, it is plausible that the interventions could have had an effect, even in the absence of a large improvement in growth, as has been reported in two other trials.^31^, ^35^ Analysis of intervention effects on micronutrient status and anaemia, parasite infection, inflammation, and environmental enteric dysfunction biomarkers will shed further light on whether there were effects on other pathways; potential mediation through any of these pathways will be examined and reported separately.

The main limitations of the study include the low uptake of some of the key promoted behaviours and the lack of data for 27% of the enrolled households by the end of the study. To examine whether losses to follow-up might have biased our findings, we did a sensitivity analysis that used inverse probability of censoring to reweight the analysis population to reflect the enrolment population based on measurable characteristics and found no substantive differences with the primary unweighted analysis. Because there were multiple groups and multiple outcome measurements, it is possible that some of the significant findings could have been due to chance. Lastly, at the time that we assessed the outcome measures, children were still fairly young. The available tests might have had too few items for each age to sensitively discern subtle developmental differences between groups in this study population. Current recommendations support the use of comprehensive assessments in children younger than 2 years for measuring concurrent abilities and identifying severe delay, but caution against using such scores to predict future development.^36^ Future follow-up might enable us to measure differences and to better understand biological pathways of effect.

In this study context, in which contacts with community health promoters were limited and in which the interventions did not have large effects on growth or diarrhoea, we noted that integrated water, sanitation, handwashing, and nutrition interventions had small effects on child gross motor development when children were aged roughly 1 year, but no effects on developmental domains at age 2 years. Future research should explore whether there are ways to make community health and nutrition programmes more effective at supporting child development, by building capacities of front-line workers to support nurturing parenting and facilitating frequent contact with caregivers. Conversely, there might be ways to incorporate water, sanitation, handwashing, or nutrition messages into child stimulation or positive parenting curricula—for example, encouraging washing the child's hands with soap or promoting responsive feeding as behaviours that foster positive caregiver–child interactions and facilitate healthy habit formation.

For **training materials** see https://osf.io/fs23x

For more on the **WHO/UNICEF Joint Monitoring Program categorisations** see https://www.wssinfo.org

For the **prespecified analysis plan** see https://osf.io/wa87d
