# Supplementary material for: Effects of water quality, sanitation, handwashing, and nutritional interventions on child development in rural Kenya (WASH Benefits Kenya): a cluster-randomised controlled trial
Source: Lancet Child Adolesc Health. 2018 Apr;2(4):269–80. doi: 10.1016/S2352-4642(18)30025-7 (PMC5859215; doi:10.1016/S2352-4642(18)30025-7)
Supplement: Supplementary appendix [file mmc1.pdf]

THE LANCET
Child & Adolescent Health

Supplementary appendix

This appendix formed part of the original submission and has been peer reviewed. We post it as supplied by the authors.

Supplement to: Stewart CP, Kariger P, Fernald L, et al. Effects of water quality, sanitation, handwashing, and nutritional interventions on child development in rural Kenya (WASH Benefits Kenya): a cluster-randomised controlled trial. *Lancet Child Adolesc Health* 2018; published online Feb 12. http://dx.doi.org/10.1016/S2352-4642(18)30025-7.

Supplementary Information for the WASH Benefits Kenya Child Development Paper

TABLE OF CONTENTS

Supplementary methods....2

Table S1: Nutrient Content of the Lipid-based Nutrient Supplement (LNS) used in WASH Benefits compared to the WHO/FAO Recommended Nutrient Intakes (RNI)[1] for children 1-3 years....3

Table S2: Uptake of target behaviors at baseline and after one and two years after the start of intervention delivery....4

Table S3: Comparison of the rate of motor milestone attainment between groups, adjusted for potential covariates.... 5

Table S4: Differences between groups in scores on the Extended Ages and Stages Questionnaire, adjusted for potential covariates.... 6

Table S5: Standardized differences in scores on the communication, gross motor, and personal social scales of the Extended Ages and Stages Questionnaire using inverse probability of censoring weighted (IPCW) analysis....7

Table S6: Effect modification with child gender....8

Table S7: Effect modification with maternal parity.... 9

Table S8: Effect modification with maternal age.... 10

Table S9: Effect modification with maternal education.... 11

Table S10: Effect modification with food insecurity....12

Table S11: Effect modification with socioeconomic status.... 13

Table S12: CONSORT Abstract checklist....14

Table S13: CONSORT Checklist (Extension for cluster designs).... 15

Supplementary methods

Extended Ages and Stages Questionnaire

The child communication, gross motor, personal-social, and global development scores were derived from the Extended Ages and Stages Questionnaire adapted from Squires and Bricker[1], which is primarily a parental report measure of child developmental progression. In addition, the child was also asked to demonstrate certain items. In the communication domain, this included: pointing to 7 or more body parts, pointing to one or more named pictures (e.g. "Show me the cow"), naming an action taking place in a picture (e.g. children sleeping, eating, playing, or reading a book). In the gross motor domain, this included: kicking a ball by swinging leg forward without holding onto anything for balance, throwing ball overhand. In the personal-social domain, this included: child recognizing self in the mirror, child offering toy to own image reflected in mirror. The item sets in the age ranges of the study children greatly overlapped, so that all children were directly observed performing at least one of the above items per domain.

Data analysis

In secondary, adjusted analyses, we pre-screened covariates to assess whether they were associated with each outcome using the likelihood ratio test. We excluded covariates that had little variation in the study population (e.g., prevalence <5%). The variables we considered included age, sex, birth order, maternal age, height and education, number of children <18 in the household, total number of people in the compound, household food insecurity, housing materials, household asset index, animal ownership, distance to water source, month of measurement, and enumerator who administered the questionnaire as potential covariates.

We also examined potential effect modification with a pre-specified list of variables including: child sex, maternal parity (first pregnancy vs. second or greater pregnancy), maternal age (<20 years old vs. ≥ 20 years old), maternal education (low education, defined as completion of primary schooling only vs. high education, defined as secondary schooling and above), household hunger (little-to-no hunger vs. moderate-to-severe hunger), and household socioeconomic status (lowest 4 quintiles vs. highest quintile). We included interaction terms in the statistical models and have presented all results stratified by category.

Because there was missing data on >20% of the enrolled study cohort, we also conducted an inverse probability of censoring-weighted analysis that reconstructs the original enrolled population.[2] This enabled us to examine if there was evidence of bias in the results due to high losses to follow-up.

References:

1. Squires J, Bricker D, Potter L. Revision of a parent-completed development screening tool: Ages and Stages Questionnaires. *J Pediatr Psychol* 1997, **22:**313-328.
2. Hernan MA, Hernandez-Diaz S, Robins JM. A structural approach to selection bias. *Epidemiology* 2004; 15(5): 615-25.

Table Supplementary Table 1. Nutrient Content of the Lipid-based Nutrient Supplement (LNS) used in WASH Benefits compared to the WHO/FAO Recommended Nutrient Intakes (RNI)[1] for children 1-3 years

			LNS Nutrient Content		
**Nutrient**	**Unit**	**RNI**	**Content**	**% RNI**	**Chemical form**
Daily Dose‡	g		20		
Energy	kcal		118		
Fat	g		9.6		
Linoleic acid	g		4.46		
Alpha-linolenic acid	g		0.58		
Ratio of LA to ALA			7.7		
Protein	g		2.6		
**Vitamins**					
Vitamin A	μg	400	400	100%	Retyinyl acetate
Vitamin D	μg	5	5	100%	Cholecalciferol (D3)
Vitamin E	mg	5	6	120%	DL-alpha-tocopherol acetate
Vitamin K	μg	15	30	200%	Phylloquinone 5%
Vitamin C	mg	30	30	100%	L-ascorbic acid
Biotin	μg	8	NA		
Folic acid	μg	150	150	100%	Pteroyl monoglutamic acid
Thiamine (B1)	mg	0.5	0.5	100%	Thiamin hydrochloride
Riboflavin (B2)	mg	0.5	0.5	100%	Riboflavin
Niacin	mg	6	6	100%	Niacinamide
Pantothenic acid (B5)	mg	2	2	100%	Calcium pantothenate
Vitamin B6	mg	0.5	0.5	100%	Pyridoxine hydrochloride
Vitamin B12	μg	0.9	0.9	100%	Cyanocobalamin (0.1%)
**Minerals**					
Calcium§	mg	500	280	56%	Tri-calcium phosphate
Copper¶	mg	0.34	0.34	100%	Encapsulated copper sulfate
Iodine	μg	90	90	100%	Potassium iodate
Iron**	mg	11.6	9	78%	Encapsulated ferrous sulfate (Bangladesh) Ferrous fumarate (Kenya) ‡‡
Magnesium§	mg	60	40	67%	Magnesium citrate
Manganese	mg	1.2	1.2	100%	Manganeze sulfate
Phosphorous§	mg	460	190	41%	Tri-calcium phosphate & Di-potassium phosphate
Potassium	mg		200		Di-potassium phosphate & potassium chloride
Selenium	μg	17	20	118%	Sodium selenite 1.5%
Zinc**	mg	8.3	8	96%	Zinc sulfate

*RNI=Recommended Nutrient Intake; LNS=Lipid-based nutrient supplement; RDA=Recommended Dietary Allowance; WHO = World Health Organization; FAO = Food and Agriculture Organization of the United Nations

‡ In malaria endemic areas, it is recommended that the supplement be split into two 10 g servings in one day to reduce the iron consumed in a single bolus dose. Although malaria is less common in Bangladesh, we recommended that children consume two 10 g sachets per day in both trials.

§ The calcium, phosphorus, and magnesium content of LNS do not meet 100% of the RNI for technical reasons

¶ The Institute of Medicine RDA level for copper for infants 1-3 y is shown here.[2]

** The RNI for iron and zinc is that assumed under a diet of low bioavailability.

‡‡ WASH Benefits Bangladesh used encapsulated ferrous sulfate, similar to other LNS products on the market. Ferrous fumarate was used in Kenya due to an interaction between ferrous sulfate and polyphenols in the commonly consumed millet flour.

References:

1. WHO and FAO, *Vitamin and Mineral Requirements in Human Nutrition*. Second Edition ed. 2004, Geneva, Switzerland: World Health Organization.
2. Institute of Medicine, *Dietary Reference Intakes: The essential guide to nutrient requirements*. 2006, Washington, DC: National Academies Press.

Supplementary Table 2: Uptake of target behaviors at baseline and after one and two years after the start of intervention delivery

	Active Control		Passive Control		Water		Sanitation		Handwashing		WSH		Nutrition		Nutrition+WSH	
	N	%	N	%	N	%	N	%	N	%	N	%	N	%	N	%
**Community Health Promoter visit in the past month**																
Baseline	0	--	0	--	0	--	0	--	0	--	0	--	0	--	0	--
Year 1	980	68	0	--	445	76	445	75	480	69	512	75	433	79	474	82
Year 2	1412	35	0	--	680	37	692	40	678	34	649	37	635	40	710	36
**Store water with detectable free chlorine**																
Baseline	1529	3	736	3	720	3	715	3	743	4	711	4	661	2	729	4
Year 1	847	3	0	--	385	39	367	5	417	5	424	42	392	2	367	43
Year 2	1365	3	0	--	637	23	641	3	648	2	598	19	614	2	652	20
**Access to improved latrine**																
Baseline	1788	17	878	17	844	18	836	16	847	19	867	18	794	15	872	16
Year 1	993	18	0	--	461	16	458	89	486	13	526	90	424	15	477	89
Year 2	1381	20	0	--	664	19	683	78	654	18	644	82	613	16	706	79
**Child feces safely disposed**																
Baseline	721	16	323	16	310	17	347	19	319	17	369	18	310	11	353	16
Year 1	903	37	0	--	424	37	412	77	431	36	463	70	391	40	432	66
Year 2	1320	10	0	--	625	8	643	37	616	10	597	34	578	9	657	33
**Primary handwashing station has water and soap**																
Baseline	1913	5	936	6	902	6	890	5	914	6	912	7	843	7	918	6
Year 1	1043	12	0	--	477	11	473	10	501	76	536	78	454	13	493	77
Year 2	1458	9	0	--	696	7	712	8	690	23	675	19	650	12	735	21
**LNS sachet consumption (% of expected)**																
Baseline	0	--	0	--	0	--	0	--	0	--	0	--	0	--	0	--
Year 1	0	--	0	--	0	--	0	--	0	--	0	--	5558	95	5838	96
Year 2	0	--	0	--	0	--	0	--	0	--	0	--	3136	114	3458	116

Free chlorine in drinking water and lipid-based nutrient supplement (LNS) consumption were not measured at enrollment and were only measured in a subset of arms. LNS adherence measured as proportion of 14 sachets consumed in the past week among index children ages 6-24 months (reported). WSH: combined water, sanitation, handwashing.

Data previously reported in Null C, Stewart CP, Pickering AJ, et al. Lancet Global Health, 2018 http://dx.doi.org/10.1016/S2214-109X(18)30005-6

Supplementary Table 3: Comparison of the rate of motor milestone attainment between groups, adjusted for potential covariates.[1]

Outcome	N	Hazard Ratio vs. Control (95% CI)	Hazard Ratio vs. WSH (95% CI)	Hazard Ratio vs. Nutrition (95% CI)
**Standing with assistance**				
Active Control	1245	Ref		
Passive Control	621	0.94 (0.81, 1.10)		
Water	607	1.13 (0.95, 1.34)		
Sanitation	585	0.96 (0.82, 1.13)		
Handwashing	605	1.03 (0.88, 1.22)		
WSH	623	1.04 (0.88, 1.23)	Ref	
Nutrition	576	1.02 (0.86, 1.20)		Ref
Nutrition + WSH	624	1.21 (1.02, 1.44)*	1.19 (0.97, 1.46)	1.23 (1.00, 1.53)*
**Walking with assistance**				
Active Control	1245	Ref		
Passive Control	621	1.02 (0.88, 1.17)		
Water	607	1.01 (0.87, 1.17)		
Sanitation	585	1.03 (0.90, 1.19)		
Handwashing	605	1.13 (0.97, 1.30)		
WSH	623	1.05 (0.91, 1.21)	Ref	
Nutrition	576	1.15 (0.98, 1.33)		Ref
Nutrition + WSH	624	1.33 (1.14, 1.54)***	1.29 (1.07, 1.54)**	1.23 (1.02, 1.48)*
**Standing alone**				
Active Control	1245	Ref		
Passive Control	621	1.10 (0.95, 1.26)		
Water	607	0.99 (0.86, 1.14)		
Sanitation	585	1.11 (0.96, 1.27)		
Handwashing	605	1.24 (1.07, 1.44)**		
WSH	623	1.03 (0.90, 1.19)	Ref	
Nutrition	576	1.10 (0.95, 1.27)		Ref
Nutrition + WSH	624	1.12 (0.97, 1.28)	1.11 (0.94, 1.31)	1.02 (0.86, 1.21)
**Walking alone**				
Active Control	1245	Ref		
Passive Control	621	1.05 (0.90, 1.22)		
Water	607	0.97 (0.82, 1.13)		
Sanitation	585	0.94 (0.80, 1.10)		
Handwashing	605	1.09 (0.93, 1.27)		
WSH	623	1.05 (0.90, 1.22)	Ref	
Nutrition	576	1.04 (0.88, 1.22)		Ref
Nutrition + WSH	624	0.93 (0.80, 1.09)	0.96 (0.80, 1.15)	0.90 (0.74, 1.08)

* $p<0.05$, ** $p<0.01$, *** $p<0.001$

[1]Potential adjustment for child age, sex, birth order, maternal age, maternal height, maternal education, number under 18y in household, number in compound, household hunger scale, floor material, roof material, household asset index, cattle ownership, goat ownership, dog ownership, poultry ownership, distance to water source, field staff interviewer, and month of measurement.

Supplementary Table 4: Differences between groups in scores on the Extended Ages and Stages Questionnaire, adjusted for potential covariates.

Outcome	N	Mean (SD)	Adjusted Difference Model 1[1]	Adjusted Difference Model 2[2]
**Communication z-score**				
Active Control	1417	0.00 (1.00)	Ref	Ref
Passive Control	656	-0.05 (0.99)	-0.05 (-0.15, 0.05)	-0.01 (-0.10, 0.07)
Water	682	-0.01 (1.01)	0.00 (-0.11, 0.11)	0.02 (-0.08, 0.12)
Sanitation	670	-0.08 (0.99)	-0.08 (-0.17, 0.02)	-0.04 (-0.12, 0.04)
Handwashing	651	-0.03 (0.99)	-0.04 (-0.15, 0.07)	-0.01 (-0.11, 0.08)
WSH	669	0.02 (0.98)	0.03 (-0.07, 0.14)	0.08 (-0.02, 0.18)
Nutrition	649	0.04 (0.99)	0.04 (-0.07, 0.15)	0.06 (-0.03, 0.15)
Nutrition + WSH	713	-0.03 (0.98)	-0.02 (-0.11, 0.08)	0.03 (-0.06, 0.12)
**Gross Motor z-score**				
Active Control	1417	0.00 (1.00)	Ref	Ref
Passive Control	656	0.00 (0.98)	0.00 (-0.11, 0.11)	0.05 (-0.03, 0.14)
Water	682	0.02 (0.99)	0.02 (-0.10, 0.13)	0.01 (-0.07, 0.10)
Sanitation	670	-0.11 (1.04)	-0.10 (-0.19, 0.00)	-0.06 (-0.14, 0.02)
Handwashing	651	-0.02 (1.03)	-0.04 (-0.16, 0.08)	0.01 (-0.09, 0.10)
WSH	669	0.02 (0.92)	0.01 (-0.10, 0.11)	0.04 (-0.05, 0.13)
Nutrition	649	0.04 (0.95)	0.02 (-0.08, 0.13)	0.06 (-0.03, 0.15)
Nutrition + WSH	713	-0.03 (0.96)	-0.03 (-0.13, 0.07)	0.02 (-0.06, 0.11)
**Personal-social z-score**				
Active Control	1417	0.00 (1.00)	Ref	Ref
Passive Control	656	0.01 (0.98)	0.00 (-0.10, 0.10)	0.01 (-0.07, 0.09)
Water	682	-0.04 (1.02)	-0.03 (-0.16, 0.09)	-0.05 (-0.15, 0.05)
Sanitation	670	-0.09 (1.01)	-0.09 (-0.20, 0.01)	-0.06 (-0.13, 0.02)
Handwashing	651	-0.04 (1.02)	-0.04 (-0.15, 0.08)	-0.02 (-0.11, 0.08)
WSH	669	-0.02 (0.98)	-0.03 (-0.13, 0.07)	-0.01 (-0.10, 0.07)
Nutrition	649	0.02 (0.96)	0.01 (-0.10, 0.12)	0.06 (-0.03, 0.14)
Nutrition + WSH	713	0.02 (0.97)	0.02 (-0.08, 0.11)	0.06 (-0.02, 0.13)
**Combined z-score**				
Active Control	1417	0.00 (1.00)	Ref	Ref
Passive Control	656	-0.02 (0.98)	-0.02 (-0.12, 0.07)	0.02 (-0.06, 0.09)
Water	682	-0.01 (1.00)	-0.01 (-0.12, 0.11)	0.00 (-0.09, 0.09)
Sanitation	670	-0.11 (1.02)	-0.11 (-0.21, -0.01)*	-0.06 (-0.14, 0.01)
Handwashing	651	-0.03 (1.01)	-0.04 (-0.16, 0.07)	-0.01 (-0.10, 0.09)
WSH	669	0.01 (0.95)	0.01 (-0.09, 0.11)	0.05 (-0.04, 0.14)
Nutrition	649	0.04 (0.98)	0.03 (-0.08, 0.15)	0.07 (-0.02, 0.16)
Nutrition + WSH	713	-0.02 (0.97)	-0.02 (-0.12, 0.08)	0.04 (-0.05, 0.13)

* $p<0.05$, ** $p<0.01$, *** $p<0.001$

[1]Potential adjustment for child age.

[2]Potential adjustment for Model 1 covariates as well as child sex, birth order, maternal age, maternal height, maternal education, number under 18y in household, number in compound, household hunger scale, floor material, roof material, household asset index, cattle ownership, goat ownership, dog ownership, poultry ownership, distance to water source, field staff interviewer, and month of measurement.

Supplementary Table 5: Standardized differences in scores on the communication, gross motor, and personal social scales of the Extended Ages and Stages Questionnaire using inverse probability of censoring weighted (IPCW) analysis.

Outcome	N	Mean (SD)	Mean Difference vs. Control (95% CI)	Mean Difference vs. WSH (95% CI)	Mean Difference vs. Nutrition (95% CI)
**Communication z-score**					
Active Control	1417	0.00 (1.00)	Ref		
Passive Control	656	-0.05 (0.99)	-0.05 (-0.15, 0.05)		
Water	682	-0.01 (1.01)	0.00 (-0.10, 0.10)		
Sanitation	670	-0.08 (0.99)	-0.09 (-0.18, 0.00)		
Handwashing	651	-0.03 (0.99)	-0.03 (-0.13, 0.07)		
WSH	669	0.02 (0.98)	0.05 (-0.05, 0.15)	Ref	
Nutrition	649	0.04 (0.99)	0.04 (-0.06, 0.15)		Ref
Nutrition + WSH	713	-0.03 (0.98)	0.00 (-0.10, 0.09)	-0.05 (-0.17, 0.06)	-0.04 (-0.15, 0.07)
**Gross Motor z-score**					
Active Control	1417	0.00 (1.00)	Ref		
Passive Control	656	0.00 (0.98)	0.01 (-0.10, 0.11)		
Water	682	0.02 (0.99)	0.02 (-0.08, 0.13)		
Sanitation	670	-0.11 (1.04)	-0.10 (-0.19, 0.00)*		
Handwashing	651	-0.02 (1.03)	-0.03 (-0.14, 0.09)		
WSH	669	0.02 (0.92)	0.03 (-0.07, 0.13)	Ref	
Nutrition	649	0.04 (0.95)	0.04 (-0.07, 0.15)		Ref
Nutrition + WSH	713	-0.03 (0.96)	-0.02 (-0.12, 0.08)	-0.04 (-0.16, 0.08)	-0.06 (-0.18, 0.06)
**Personal-social z-score**					
Active Control	1417	0.00 (1.00)	Ref		
Passive Control	656	0.01 (0.98)	-0.02 (-0.12, 0.09)		
Water	682	-0.04 (1.02)	-0.03 (-0.15, 0.09)		
Sanitation	670	-0.09 (1.01)	-0.09 (-0.19, 0.01)		
Handwashing	651	-0.04 (1.02)	-0.03 (-0.14, 0.07)		
WSH	669	-0.02 (0.98)	-0.03 (-0.13, 0.07)	Ref	
Nutrition	649	0.02 (0.96)	0.00 (-0.10, 0.11)		Ref
Nutrition + WSH	713	0.02 (0.97)	0.03 (-0.07, 0.12)	0.06 (-0.05, 0.17)	0.02 (-0.09, 0.13)
**Combined z-score**					
Active Control	1417	0.00 (1.00)	Ref		
Passive Control	656	-0.02 (0.98)	-0.03 (-0.12, 0.07)		
Water	682	-0.01 (1.00)	0.00 (-0.10, 0.10)		
Sanitation	670	-0.11 (1.02)	-0.11 (-0.20, -0.02)*		
Handwashing	651	-0.03 (1.01)	-0.03 (-0.15, 0.08)		
WSH	669	0.01 (0.95)	0.02 (-0.07, 0.12)	Ref	
Nutrition	649	0.04 (0.98)	0.04 (-0.07, 0.15)		Ref
Nutrition + WSH	713	-0.02 (0.97)	-0.01 (-0.10, 0.09)	-0.02 (-0.14, 0.10)	-0.05 (-0.17, 0.07)

* p<0.05, ** p<0.01, *** p<0.001

Supplementary Table 6: Effect modification with child gender

	Male			Female			
**Outcome**	**N**	**Mean (SD)**	**Mean Difference vs. Control (95% CI)**	**N**	**Mean (SD)**	**Mean Difference vs. Control (95% CI)**	**P for Interaction**
**Communication z-score**							
Active Control	679	-0.08 (1.04)		738	0.08 (0.95)		
Passive Control	301	-0.16 (1.04)	-0.06 (-0.21, 0.09)	355	0.05 (0.94)	0.00 (-0.11, 0.12)	0.462
Water	327	-0.03 (1.05)	0.06 (-0.08, 0.20)	355	0.01 (0.97)	-0.03 (-0.16, 0.10)	0.319
Sanitation	322	-0.18 (1.03)	-0.04 (-0.18, 0.09)	348	0.00 (0.95)	-0.03 (-0.13, 0.08)	0.862
Handwashing	316	-0.15 (0.99)	-0.08 (-0.22, 0.06)	335	0.08 (0.97)	-0.02 (-0.15, 0.11)	0.569
WSH	315	-0.06 (1.00)	0.04 (-0.11, 0.19)	354	0.09 (0.96)	0.06 (-0.06, 0.18)	0.807
Nutrition	340	-0.06 (1.00)	0.01 (-0.12, 0.14)	309	0.14 (0.96)	0.09 (-0.04, 0.22)	0.326
Nutrition + WSH	342	-0.16 (1.02)	-0.04 (-0.15, 0.08)	371	0.09 (0.92)	0.04 (-0.09, 0.18)	0.388
N + WSH vs WSH			-0.09 (-0.24, 0.06)			-0.05 (-0.19, 0.09)	0.661
N + WSH vs N			-0.05 (-0.21, 0.10)			-0.09 (-0.23, 0.05)	0.715
**Gross Motor z-score**							
Active Control	679	-0.01 (0.99)		738	0.01 (1.01)		
Passive Control	301	0.02 (0.97)	0.07 (-0.08, 0.21)	355	-0.02 (0.99)	-0.01 (-0.15, 0.13)	0.479
Water	327	-0.03 (1.02)	-0.03 (-0.15, 0.10)	355	0.06 (0.95)	0.11 (-0.04, 0.25)	0.173
Sanitation	322	-0.14 (1.05)	-0.10 (-0.23, 0.03)	348	-0.07 (1.02)	-0.02 (-0.16, 0.11)	0.440
Handwashing	316	-0.07 (1.02)	-0.06 (-0.22, 0.10)	335	0.01 (1.04)	0.01 (-0.13, 0.15)	0.495
WSH	315	-0.06 (0.94)	-0.05 (-0.20, 0.09)	354	0.08 (0.91)	0.08 (-0.06, 0.23)	0.202
Nutrition	340	0.06 (0.95)	0.04 (-0.08, 0.16)	309	0.01 (0.96)	0.02 (-0.10, 0.15)	0.814
Nutrition + WSH	342	-0.07 (1.02)	-0.05 (-0.20, 0.10)	371	0.00 (0.90)	0.03 (-0.11, 0.17)	0.453
N + WSH vs WSH			-0.01 (-0.16, 0.15)			-0.06 (-0.23, 0.10)	0.598
N + WSH vs N			-0.07 (-0.23, 0.09)			-0.02 (-0.19, 0.16)	0.637
**Personal-social z-score**							
Active Control	679	-0.11 (1.00)		738	0.10 (0.99)		
Passive Control	301	-0.07 (1.02)	0.04 (-0.09, 0.16)	355	0.08 (0.93)	-0.01 (-0.12, 0.10)	0.540
Water	327	-0.13 (1.08)	0.00 (-0.15, 0.16)	355	0.05 (0.95)	-0.03 (-0.15, 0.09)	0.691
Sanitation	322	-0.20 (0.99)	-0.04 (-0.19, 0.11)	348	0.01 (1.02)	-0.06 (-0.18, 0.06)	0.836
Handwashing	316	-0.19 (1.01)	-0.08 (-0.23, 0.07)	335	0.11 (1.01)	-0.03 (-0.15, 0.09)	0.648
WSH	315	-0.12 (1.00)	0.01 (-0.13, 0.15)	354	0.07 (0.95)	-0.03 (-0.16, 0.09)	0.669
Nutrition	340	-0.06 (1.00)	0.04 (-0.10, 0.18)	309	0.11 (0.92)	-0.01 (-0.15, 0.12)	0.538
Nutrition + WSH	342	-0.16 (1.03)	-0.04 (-0.17, 0.09)	371	0.18 (0.88)	0.07 (-0.04, 0.19)	0.227
N + WSH vs WSH			-0.04 (-0.20, 0.11)			0.08 (-0.08, 0.24)	0.229
N + WSH vs N			-0.09 (-0.25, 0.07)			0.09 (-0.07, 0.24)	0.111
**Combined z-score**							
Active Control	679	-0.08 (1.02)		738	0.07 (0.97)		
Passive Control	301	-0.09 (1.03)	0.01 (-0.12, 0.14)	355	0.04 (0.94)	-0.01 (-0.13, 0.11)	0.827
Water	327	-0.07 (1.07)	0.02 (-0.12, 0.15)	355	0.05 (0.94)	0.02 (-0.12, 0.15)	0.991
Sanitation	322	-0.21 (1.03)	-0.07 (-0.21, 0.07)	348	-0.03 (0.99)	-0.05 (-0.16, 0.07)	0.770
Handwashing	316	-0.16 (1.01)	-0.09 (-0.24, 0.06)	335	0.08 (1.00)	-0.01 (-0.14, 0.11)	0.466
WSH	315	-0.09 (0.98)	0.00 (-0.15, 0.15)	354	0.10 (0.92)	0.05 (-0.08, 0.18)	0.606
Nutrition	340	-0.02 (0.99)	0.04 (-0.10, 0.17)	309	0.11 (0.96)	0.05 (-0.08, 0.18)	0.868
Nutrition + WSH	342	-0.16 (1.04)	-0.05 (-0.19, 0.09)	371	0.10 (0.89)	0.06 (-0.07, 0.18)	0.266
N + WSH vs WSH			-0.06 (-0.22, 0.09)			-0.03 (-0.18, 0.13)	0.723
N + WSH vs N			-0.09 (-0.25, 0.07)			-0.02 (-0.18, 0.13)	0.539

* $p<0.05$, ** $p<0.01$, *** $p<0.001$

Supplementary Table 7: Effect modification with maternal parity

	Multiparous			Nulliparous			
**Outcome**	**N**	**Mean (SD)**	**Mean Difference vs. Control (95% CI)**	**N**	**Mean (SD)**	**Mean Difference vs. Control (95% CI)**	**P for Interaction**
**Communication z-score**							
Active Control	310	-0.04 (1.05)		1103	0.01 (0.98)		
Passive Control	138	-0.02 (1.00)	0.01 (-0.18, 0.20)	518	-0.06 (0.99)	-0.03 (-0.15, 0.08)	0.680
Water	131	-0.14 (1.11)	-0.12 (-0.38, 0.14)	551	0.02 (0.98)	0.04 (-0.08, 0.15)	0.268
Sanitation	151	0.07 (0.92)	0.13 (-0.08, 0.34)	519	-0.13 (1.01)	-0.08 (-0.19, 0.02)	0.093
Handwashing	129	-0.14 (1.03)	-0.13 (-0.34, 0.09)	522	0.00 (0.97)	-0.04 (-0.14, 0.07)	0.445
WSH	115	0.03 (0.96)	0.10 (-0.11, 0.30)	554	0.02 (0.99)	0.04 (-0.09, 0.16)	0.652
Nutrition	143	0.12 (0.94)	0.18 (-0.02, 0.38)	506	0.01 (1.00)	0.00 (-0.13, 0.13)	0.154
Nutrition + WSH	146	-0.09 (0.94)	-0.06 (-0.26, 0.14)	565	-0.01 (0.99)	0.02 (-0.08, 0.12)	0.449
N + WSH vs WSH			-0.13 (-0.38, 0.11)			-0.06 (-0.19, 0.07)	0.617
N + WSH vs N			-0.31 (-0.57, -0.06)*			0.00 (-0.11, 0.12)	
**Gross Motor z-score**							
Active Control	310	0.10 (0.94)		1103	-0.02 (1.01)		
Passive Control	138	0.13 (0.84)	0.01 (-0.15, 0.18)	518	-0.04 (1.02)	0.03 (-0.09, 0.14)	0.890
Water	131	-0.04 (1.01)	-0.10 (-0.32, 0.11)	551	0.03 (0.98)	0.07 (-0.04, 0.19)	0.153
Sanitation	151	-0.01 (1.04)	-0.05 (-0.29, 0.18)	519	-0.13 (1.04)	-0.07 (-0.17, 0.04)	0.925
Handwashing	129	0.02 (1.03)	-0.05 (-0.27, 0.17)	522	-0.04 (1.04)	-0.02 (-0.14, 0.10)	0.800
WSH	115	0.15 (0.85)	0.03 (-0.15, 0.22)	554	-0.01 (0.94)	0.02 (-0.09, 0.13)	0.905
Nutrition	143	0.07 (0.93)	-0.04 (-0.27, 0.18)	506	0.03 (0.96)	0.05 (-0.06, 0.16)	0.460
Nutrition + WSH	146	0.07 (1.01)	0.01 (-0.22, 0.24)	565	-0.06 (0.94)	-0.01 (-0.12, 0.09)	0.878
N + WSH vs WSH			-0.04 (-0.28, 0.20)			-0.04 (-0.16, 0.09)	0.948
N + WSH vs N			0.03 (-0.20, 0.25)			-0.06 (-0.19, 0.08)	0.496
**Personal-social z-score**							
Active Control	310	-0.02 (1.00)		1103	0.01 (1.00)		
Passive Control	138	0.00 (0.93)	0.02 (-0.16, 0.20)	518	0.01 (0.99)	0.01 (-0.09, 0.12)	0.961
Water	131	-0.08 (1.06)	-0.03 (-0.27, 0.21)	551	-0.02 (1.01)	-0.02 (-0.14, 0.10)	0.945
Sanitation	151	0.01 (1.01)	0.05 (-0.16, 0.25)	519	-0.12 (1.01)	-0.08 (-0.20, 0.03)	0.257
Handwashing	129	-0.01 (1.07)	-0.01 (-0.24, 0.23)	522	-0.04 (1.01)	-0.07 (-0.18, 0.04)	0.655
WSH	115	0.08 (1.05)	0.12 (-0.12, 0.35)	554	-0.04 (0.96)	-0.04 (-0.15, 0.07)	0.254
Nutrition	143	0.00 (0.92)	0.02 (-0.20, 0.25)	506	0.03 (0.98)	0.00 (-0.14, 0.13)	0.850
Nutrition + WSH	146	0.12 (0.96)	0.16 (-0.06, 0.38)	565	-0.01 (0.97)	-0.02 (-0.12, 0.08)	0.165
N + WSH vs WSH			0.01 (-0.27, 0.30)			0.01 (-0.12, 0.15)	0.997
N + WSH vs N			0.04 (-0.20, 0.28)			0.00 (-0.13, 0.13)	0.755
**Combined z-score**							
Active Control	310	0.02 (0.97)		1103	0.00 (1.00)		
Passive Control	138	0.05 (0.91)	0.02 (-0.15, 0.18)	518	-0.04 (1.00)	0.00 (-0.10, 0.10)	0.839
Water	131	-0.11 (1.06)	-0.12 (-0.35, 0.12)	551	0.01 (0.99)	0.04 (-0.06, 0.15)	0.208
Sanitation	151	0.02 (0.97)	0.05 (-0.16, 0.26)	519	-0.15 (1.03)	-0.09 (-0.20, 0.01)	0.253
Handwashing	129	-0.06 (1.07)	-0.08 (-0.31, 0.14)	522	-0.03 (1.00)	-0.04 (-0.15, 0.06)	0.768
WSH	115	0.11 (0.94)	0.10 (-0.11, 0.30)	554	-0.01 (0.95)	0.01 (-0.10, 0.13)	0.484
Nutrition	143	0.08 (0.93)	0.07 (-0.15, 0.29)	506	0.03 (0.99)	0.02 (-0.10, 0.15)	0.714
Nutrition + WSH	146	0.02 (0.97)	0.02 (-0.21, 0.24)	565	-0.03 (0.97)	0.00 (-0.10, 0.10)	0.889
N + WSH vs WSH			-0.08 (-0.33, 0.16)			-0.04 (-0.17, 0.09)	0.752
N + WSH vs N			-0.13 (-0.38, 0.13)			-0.03 (-0.15, 0.10)	0.449

* p<0.05, ** p<0.01, *** p<0.001

Supplementary Table 8: Effect modification with maternal age

	**Mother over 20y old**			**Mother 20y old or younger**			
**Outcome**	**N**	**Mean (SD)**	**Mean Difference vs. Control (95% CI)**	**N**	**Mean (SD)**	**Mean Difference vs. Control (95% CI)**	**P for Interaction**
**Communication z-score**							
Active Control	1161	0.05 (0.98)		244	-0.20 (1.04)		
Passive Control	539	-0.06 (1.02)	-0.06 (-0.17, 0.05)	111	0.01 (0.85)	0.18 (-0.02, 0.37)	
Water	586	0.01 (0.99)	0.00 (-0.11, 0.11)	92	-0.13 (1.11)	0.01 (-0.30, 0.32)	0.961
Sanitation	534	-0.08 (1.00)	-0.07 (-0.17, 0.04)	134	-0.11 (0.99)	0.11 (-0.11, 0.33)	0.183
Handwashing	543	-0.01 (0.98)	-0.07 (-0.17, 0.03)	104	-0.14 (1.00)	0.02 (-0.17, 0.22)	0.376
WSH	565	0.03 (0.98)	0.02 (-0.09, 0.13)	101	-0.03 (0.98)	0.19 (-0.04, 0.42)	0.189
Nutrition	535	0.06 (0.98)	0.03 (-0.08, 0.15)	108	-0.08 (1.03)	0.10 (-0.12, 0.32)	0.568
Nutrition + WSH	587	0.01 (0.97)	0.01 (-0.10, 0.12)	117	-0.17 (1.01)	0.00 (-0.19, 0.20)	0.959
N + WSH vs WSH			-0.04 (-0.17, 0.08)			-0.20 (-0.47, 0.07)	0.328
N + WSH vs N			-0.05 (-0.16, 0.06)			-0.12 (-0.41, 0.17)	0.608
**Gross Motor z-score**							
Active Control	1161	0.01 (1.01)		244	-0.06 (0.94)		
Passive Control	539	0.00 (1.00)	0.03 (-0.08, 0.13)	111	-0.02 (0.92)	0.03 (-0.20, 0.25)	0.989
Water	586	0.05 (0.97)	0.07 (-0.04, 0.17)	92	-0.22 (1.06)	-0.16 (-0.41, 0.09)	0.098
Sanitation	534	-0.10 (1.04)	-0.07 (-0.17, 0.02)	134	-0.12 (1.04)	-0.02 (-0.25, 0.20)	0.681
Handwashing	543	-0.03 (1.03)	-0.05 (-0.16, 0.07)	104	-0.01 (1.06)	0.06 (-0.16, 0.27)	0.397
WSH	565	0.01 (0.94)	0.00 (-0.11, 0.11)	101	0.07 (0.86)	0.11 (-0.11, 0.34)	0.353
Nutrition	535	0.09 (0.94)	0.07 (-0.03, 0.17)	108	-0.18 (1.00)	-0.15 (-0.35, 0.05)	
Nutrition + WSH	587	-0.04 (0.94)	-0.03 (-0.13, 0.07)	117	0.01 (1.04)	0.08 (-0.15, 0.31)	0.391
N + WSH vs WSH			-0.03 (-0.15, 0.10)			-0.08 (-0.35, 0.19)	0.709
N + WSH vs N			-0.10 (-0.23, 0.04)			0.21 (-0.07, 0.49)	
**Personal-social z-score**							
Active Control	1161	0.03 (0.99)		244	-0.12 (1.03)		
Passive Control	539	0.02 (1.00)	0.00 (-0.10, 0.11)	111	-0.01 (0.90)	0.07 (-0.16, 0.30)	0.613
Water	586	-0.01 (1.02)	-0.02 (-0.12, 0.08)	92	-0.19 (1.01)	-0.09 (-0.39, 0.20)	0.593
Sanitation	534	-0.10 (1.02)	-0.08 (-0.19, 0.03)	134	-0.08 (0.96)	0.07 (-0.18, 0.31)	0.285
Handwashing	543	-0.05 (1.03)	-0.09 (-0.19, 0.01)	104	0.04 (0.97)	0.13 (-0.14, 0.40)	0.134
WSH	565	-0.04 (0.96)	-0.05 (-0.15, 0.05)	101	0.06 (1.03)	0.18 (-0.08, 0.45)	0.106
Nutrition	535	0.06 (0.95)	0.02 (-0.09, 0.14)	108	-0.17 (0.97)	-0.07 (-0.33, 0.18)	0.496
Nutrition + WSH	587	0.03 (0.94)	0.02 (-0.08, 0.12)	117	-0.03 (1.09)	0.08 (-0.16, 0.32)	0.636
N + WSH vs WSH			0.06 (-0.06, 0.18)			-0.18 (-0.46, 0.10)	0.089
N + WSH vs N			-0.01 (-0.13, 0.10)			0.12 (-0.17, 0.42)	0.364
**Combined z-score**							
Active Control	1161	0.04 (0.99)		244	-0.16 (0.98)		
Passive Control	539	-0.02 (1.00)	-0.02 (-0.12, 0.08)	111	0.00 (0.90)	0.12 (-0.09, 0.32)	0.259
Water	586	0.02 (0.99)	0.02 (-0.08, 0.12)	92	-0.21 (1.09)	-0.09 (-0.38, 0.19)	0.429
Sanitation	534	-0.11 (1.02)	-0.09 (-0.19, 0.01)	134	-0.13 (0.99)	0.06 (-0.17, 0.29)	0.250
Handwashing	543	-0.03 (1.02)	-0.08 (-0.18, 0.02)	104	-0.05 (1.01)	0.08 (-0.13, 0.29)	0.164
WSH	565	0.01 (0.95)	-0.01 (-0.11, 0.10)	101	0.03 (0.96)	0.19 (-0.04, 0.43)	0.116
Nutrition	535	0.09 (0.97)	0.05 (-0.06, 0.17)	108	-0.17 (1.00)	-0.04 (-0.26, 0.18)	0.438
Nutrition + WSH	587	0.00 (0.95)	0.00 (-0.10, 0.10)	117	-0.09 (1.06)	0.06 (-0.16, 0.28)	0.638
N + WSH vs WSH			-0.01 (-0.14, 0.11)			-0.19 (-0.46, 0.09)	0.246
N + WSH vs N			-0.07 (-0.19, 0.05)			0.07 (-0.22, 0.36)	0.334

* p<0.05, ** p<0.01, *** p<0.001

Supplementary Table 9: Effect modification with maternal education

	Low Education			High Education			
**Outcome**	**N**	**Mean (SD)**	**Mean Difference vs. Control (95% CI)**	**N**	**Mean (SD)**	**Mean Difference vs. Control (95% CI)**	**P for Interaction**
**Communication z-score**							
Active Control	1274	-0.04 (1.00)		140	0.40 (0.87)		
Passive Control	571	-0.10 (0.99)	-0.02 (-0.12, 0.09)	85	0.26 (0.97)	-0.19 (-0.46, 0.08)	0.234
Water	600	-0.06 (1.03)	0.01 (-0.11, 0.13)	82	0.36 (0.75)	-0.09 (-0.30, 0.12)	0.451
Sanitation	585	-0.13 (0.99)	-0.04 (-0.13, 0.05)	85	0.21 (0.96)	-0.11 (-0.41, 0.20)	0.666
Handwashing	576	-0.08 (1.00)	-0.06 (-0.16, 0.04)	75	0.38 (0.80)	-0.02 (-0.30, 0.27)	0.771
WSH	600	-0.04 (1.00)	0.04 (-0.07, 0.15)	69	0.55 (0.60)	0.15 (-0.09, 0.39)	0.420
Nutrition	574	-0.03 (1.00)	0.01 (-0.10, 0.13)	75	0.51 (0.74)	0.21 (-0.06, 0.48)	0.183
Nutrition + WSH	633	-0.05 (0.98)	0.03 (-0.06, 0.11)	78	0.14 (0.96)	-0.23 (-0.52, 0.06)	0.090
N + WSH vs WSH			-0.05 (-0.15, 0.05)			-0.33 (-0.62, -0.05)*	
N + WSH vs N			-0.02 (-0.15, 0.10)			-0.41 (-0.70, -0.11)*	
**Gross Motor z-score**							
Active Control	1274	-0.03 (1.01)		140	0.33 (0.81)		
Passive Control	571	-0.06 (1.00)	0.01 (-0.10, 0.12)	85	0.39 (0.76)	0.01 (-0.21, 0.23)	0.995
Water	600	-0.02 (0.99)	0.05 (-0.05, 0.15)	82	0.24 (0.92)	-0.11 (-0.38, 0.16)	0.250
Sanitation	585	-0.13 (1.05)	-0.06 (-0.16, 0.04)	85	0.10 (0.95)	-0.17 (-0.43, 0.08)	0.425
Handwashing	576	-0.06 (1.04)	-0.02 (-0.14, 0.09)	75	0.21 (0.94)	-0.11 (-0.41, 0.19)	0.600
WSH	600	-0.01 (0.94)	0.02 (-0.08, 0.13)	69	0.29 (0.76)	-0.05 (-0.26, 0.16)	0.532
Nutrition	574	-0.01 (0.97)	0.02 (-0.09, 0.13)	75	0.37 (0.75)	0.09 (-0.16, 0.34)	0.629
Nutrition + WSH	633	-0.05 (0.96)	0.00 (-0.10, 0.10)	78	0.11 (0.90)	-0.16 (-0.40, 0.09)	0.231
N + WSH vs WSH			-0.03 (-0.15, 0.09)			-0.13 (-0.42, 0.16)	0.515
N + WSH vs N			-0.02 (-0.15, 0.11)			-0.22 (-0.47, 0.03)	0.143
**Personal-social z-score**							
Active Control	1274	-0.03 (1.01)		140	0.29 (0.85)		
Passive Control	571	-0.02 (1.00)	0.02 (-0.08, 0.12)	85	0.25 (0.80)	-0.07 (-0.28, 0.15)	0.490
Water	600	-0.07 (1.03)	-0.02 (-0.14, 0.10)	82	0.25 (0.91)	-0.07 (-0.33, 0.19)	0.714
Sanitation	585	-0.14 (1.01)	-0.07 (-0.17, 0.03)	85	0.23 (0.92)	0.00 (-0.28, 0.29)	0.594
Handwashing	576	-0.08 (1.03)	-0.07 (-0.18, 0.03)	75	0.34 (0.84)	0.06 (-0.17, 0.29)	0.307
WSH	600	-0.05 (0.99)	-0.01 (-0.11, 0.10)	69	0.22 (0.81)	-0.05 (-0.32, 0.22)	0.777
Nutrition	574	-0.02 (0.98)	-0.01 (-0.13, 0.11)	75	0.34 (0.79)	0.11 (-0.13, 0.34)	0.389
Nutrition + WSH	633	0.01 (0.97)	0.03 (-0.05, 0.12)	78	0.08 (1.00)	-0.14 (-0.40, 0.13)	0.212
N + WSH vs WSH			0.02 (-0.10, 0.15)			-0.08 (-0.39, 0.24)	0.551
N + WSH vs N			0.03 (-0.09, 0.15)			-0.20 (-0.49, 0.10)	0.167
**Combined z-score**							
Active Control	1274	-0.04 (1.00)		140	0.42 (0.83)		
Passive Control	571	-0.08 (0.99)	0.00 (-0.09, 0.10)	85	0.37 (0.82)	-0.11 (-0.34, 0.12)	0.400
Water	600	-0.06 (1.02)	0.02 (-0.09, 0.13)	82	0.35 (0.81)	-0.11 (-0.34, 0.11)	0.303
Sanitation	585	-0.16 (1.02)	-0.07 (-0.16, 0.03)	85	0.22 (0.91)	-0.12 (-0.41, 0.17)	0.733
Handwashing	576	-0.09 (1.02)	-0.06 (-0.16, 0.04)	75	0.38 (0.84)	-0.03 (-0.29, 0.24)	0.815
WSH	600	-0.04 (0.97)	0.03 (-0.08, 0.13)	69	0.46 (0.61)	0.04 (-0.19, 0.27)	0.920
Nutrition	574	-0.02 (0.99)	0.01 (-0.11, 0.13)	75	0.52 (0.73)	0.18 (-0.07, 0.43)	0.231
Nutrition + WSH	633	-0.04 (0.97)	0.02 (-0.06, 0.11)	78	0.14 (0.94)	-0.21 (-0.49, 0.06)	0.092
N + WSH vs WSH			-0.03 (-0.15, 0.09)			-0.24 (-0.52, 0.04)	0.138
N + WSH vs N			-0.02 (-0.15, 0.12)			-0.35 (-0.61, -0.09)*	

* p<0.05, ** p<0.01, *** p<0.001

Supplementary Table 10: Effect modification with food insecurity

	Little to no hunger			Moderate to severe hunger			
**Outcome**	**N**	**Mean (SD)**	**Mean Difference vs. Control (95% CI)**	**N**	**Mean (SD)**	**Mean Difference vs. Control (95% CI)**	**P for Interaction**
**Communication z-score**							
Active Control	1263	0.01 (0.99)		145	-0.14 (1.03)		
Passive Control	587	-0.03 (0.99)	-0.02 (-0.13, 0.08)	68	-0.23 (1.03)	-0.03 (-0.30, 0.25)	0.984
Water	603	0.00 (1.01)	0.00 (-0.10, 0.11)	79	-0.04 (0.96)	0.08 (-0.26, 0.41)	0.681
Sanitation	606	-0.08 (1.00)	-0.04 (-0.14, 0.06)	64	-0.14 (0.92)	0.01 (-0.30, 0.31)	0.793
Handwashing	585	-0.01 (0.98)	-0.04 (-0.15, 0.06)	64	-0.16 (0.99)	-0.06 (-0.35, 0.23)	0.909
WSH	600	0.04 (0.98)	0.05 (-0.05, 0.16)	67	-0.13 (1.01)	0.03 (-0.29, 0.35)	0.910
Nutrition	581	0.05 (0.98)	0.04 (-0.08, 0.15)	68	-0.09 (1.07)	0.07 (-0.28, 0.42)	0.854
Nutrition + WSH	633	-0.01 (0.96)	0.01 (-0.09, 0.11)	78	-0.14 (1.11)	0.01 (-0.28, 0.29)	0.983
N + WSH vs WSH			-0.08 (-0.19, 0.04)			-0.06 (-0.42, 0.30)	0.934
N + WSH vs N			-0.06 (-0.19, 0.06)			-0.04 (-0.41, 0.34)	0.894
**Gross Motor z-score**							
Active Control	1263	0.01 (0.99)		145	-0.10 (1.03)		
Passive Control	587	0.01 (0.98)	0.02 (-0.09, 0.12)	68	-0.06 (1.00)	0.09 (-0.23, 0.41)	0.679
Water	603	0.03 (0.98)	0.05 (-0.05, 0.14)	79	-0.06 (1.02)	-0.02 (-0.33, 0.29)	0.672
Sanitation	606	-0.09 (1.03)	-0.06 (-0.16, 0.04)	64	-0.23 (1.08)	-0.07 (-0.33, 0.18)	0.934
Handwashing	585	0.00 (1.02)	-0.01 (-0.13, 0.10)	64	-0.24 (1.16)	-0.12 (-0.44, 0.19)	0.546
WSH	600	0.04 (0.92)	0.03 (-0.09, 0.14)	67	-0.18 (0.95)	-0.05 (-0.36, 0.26)	0.672
Nutrition	581	0.07 (0.94)	0.04 (-0.07, 0.14)	68	-0.19 (1.03)	0.01 (-0.30, 0.31)	0.869
Nutrition + WSH	633	-0.03 (0.96)	-0.01 (-0.11, 0.09)	78	-0.02 (0.95)	0.04 (-0.26, 0.35)	0.735
N + WSH vs WSH			-0.06 (-0.18, 0.06)			0.16 (-0.20, 0.51)	0.250
N + WSH vs N			-0.05 (-0.18, 0.08)			0.06 (-0.19, 0.30)	0.403
**Personal-social z-score**							
Active Control	1263	0.01 (0.99)		145	-0.15 (1.10)		
Passive Control	587	0.02 (0.97)	0.00 (-0.09, 0.10)	68	-0.05 (1.02)	0.13 (-0.15, 0.41)	0.401
Water	603	-0.03 (1.02)	-0.03 (-0.14, 0.08)	79	-0.05 (1.03)	0.08 (-0.25, 0.41)	0.516
Sanitation	606	-0.08 (1.01)	-0.06 (-0.17, 0.05)	64	-0.21 (0.95)	-0.01 (-0.29, 0.26)	0.786
Handwashing	585	-0.02 (1.03)	-0.05 (-0.16, 0.05)	64	-0.17 (0.97)	-0.08 (-0.37, 0.21)	0.852
WSH	600	-0.02 (0.97)	-0.03 (-0.12, 0.07)	67	-0.07 (1.03)	0.13 (-0.19, 0.46)	0.346
Nutrition	581	0.05 (0.95)	0.00 (-0.11, 0.12)	68	-0.19 (1.06)	0.01 (-0.33, 0.36)	0.956
Nutrition + WSH	633	0.02 (0.96)	0.02 (-0.07, 0.11)	78	-0.05 (1.07)	0.04 (-0.27, 0.36)	0.890
N + WSH vs WSH			0.04 (-0.09, 0.16)			-0.13 (-0.51, 0.24)	0.396
N + WSH vs N			-0.01 (-0.13, 0.11)			0.13 (-0.22, 0.47)	0.467
**Combined z-score**							
Active Control	1263	0.02 (0.99)		145	-0.16 (1.06)		
Passive Control	587	-0.01 (0.98)	0.00 (-0.10, 0.09)	68	-0.15 (0.97)	0.07 (-0.19, 0.33)	0.613
Water	603	0.00 (1.00)	0.01 (-0.09, 0.11)	79	-0.06 (1.03)	0.06 (-0.30, 0.41)	0.803
Sanitation	606	-0.10 (1.02)	-0.06 (-0.17, 0.04)	64	-0.24 (0.99)	-0.03 (-0.31, 0.25)	0.846
Handwashing	585	-0.01 (1.01)	-0.04 (-0.14, 0.06)	64	-0.23 (1.04)	-0.10 (-0.40, 0.19)	0.702
WSH	600	0.03 (0.95)	0.03 (-0.08, 0.13)	67	-0.16 (1.01)	0.04 (-0.28, 0.37)	0.929
Nutrition	581	0.07 (0.96)	0.04 (-0.08, 0.15)	68	-0.18 (1.11)	0.05 (-0.29, 0.39)	0.959
Nutrition + WSH	633	-0.01 (0.96)	0.00 (-0.09, 0.10)	78	-0.09 (1.06)	0.04 (-0.24, 0.32)	0.812
N + WSH vs WSH			-0.05 (-0.17, 0.07)			0.00 (-0.37, 0.37)	0.813
N + WSH vs N			-0.06 (-0.19, 0.07)			0.05 (-0.25, 0.36)	0.515

* p<0.05, ** p<0.01, *** p<0.001

Supplementary Table 11: Effect modification with socioeconomic status

	Low quintiles			Highest quintile			
**Outcome**	**N**	**Mean (SD)**	**Mean Difference vs. Control (95% CI)**	**N**	**Mean (SD)**	**Mean Difference vs. Control (95% CI)**	**P for Interaction**
**Communication z-score**							
Active Control	1146	-0.05 (1.01)		268	0.22 (0.89)		
Passive Control	530	-0.08 (0.99)	-0.02 (-0.13, 0.09)	126	0.07 (0.99)	-0.06 (-0.30, 0.17)	0.729
Water	532	-0.08 (1.03)	-0.02 (-0.15, 0.11)	150	0.25 (0.89)	0.08 (-0.09, 0.24)	0.350
Sanitation	532	-0.12 (1.00)	-0.03 (-0.13, 0.06)	138	0.04 (0.95)	-0.06 (-0.24, 0.11)	0.752
Handwashing	508	-0.06 (0.98)	-0.06 (-0.17, 0.05)	143	0.08 (0.99)	-0.05 (-0.24, 0.14)	0.952
WSH	549	-0.02 (0.99)	0.04 (-0.07, 0.14)	120	0.22 (0.89)	0.12 (-0.06, 0.31)	0.414
Nutrition	512	-0.01 (1.01)	0.03 (-0.09, 0.15)	137	0.19 (0.86)	0.05 (-0.14, 0.24)	0.862
Nutrition + WSH	563	-0.08 (0.99)	-0.01 (-0.11, 0.10)	148	0.16 (0.92)	0.03 (-0.14, 0.21)	0.690
N + WSH vs WSH			-0.09 (-0.20, 0.03)			-0.07 (-0.30, 0.15)	0.920
N + WSH vs N			-0.06 (-0.19, 0.06)			-0.06 (-0.29, 0.18)	0.962
**Gross Motor z-score**							
Active Control	1146	-0.04 (0.99)		268	0.18 (0.97)		
Passive Control	530	-0.02 (0.98)	0.03 (-0.07, 0.14)	126	0.08 (0.98)	-0.03 (-0.21, 0.14)	0.491
Water	532	-0.03 (1.01)	0.03 (-0.09, 0.15)	150	0.19 (0.89)	0.05 (-0.14, 0.24)	0.876
Sanitation	532	-0.13 (1.06)	-0.06 (-0.16, 0.03)	138	-0.02 (0.96)	-0.08 (-0.28, 0.11)	0.859
Handwashing	508	0.01 (1.00)	0.03 (-0.09, 0.14)	143	-0.14 (1.14)	-0.24 (-0.48, 0.00)*	
WSH	549	0.00 (0.94)	0.03 (-0.08, 0.13)	120	0.11 (0.84)	-0.02 (-0.23, 0.18)	0.636
Nutrition	512	0.02 (0.96)	0.05 (-0.06, 0.16)	137	0.11 (0.91)	-0.07 (-0.28, 0.15)	0.361
Nutrition + WSH	563	-0.06 (0.97)	-0.01 (-0.12, 0.10)	148	0.07 (0.91)	-0.03 (-0.21, 0.15)	0.889
N + WSH vs WSH			-0.05 (-0.17, 0.08)			-0.01 (-0.24, 0.22)	0.783
N + WSH vs N			-0.06 (-0.20, 0.08)			0.02 (-0.21, 0.26)	0.569
**Personal-social z-score**							
Active Control	1146	-0.02 (1.00)		268	0.10 (0.99)		
Passive Control	530	0.02 (0.97)	0.04 (-0.06, 0.14)	126	-0.01 (1.01)	-0.08 (-0.32, 0.16)	0.372
Water	532	-0.11 (1.02)	-0.07 (-0.20, 0.05)	150	0.23 (0.95)	0.17 (-0.02, 0.36)	
Sanitation	532	-0.12 (1.01)	-0.07 (-0.18, 0.04)	138	0.02 (1.01)	0.01 (-0.20, 0.22)	0.471
Handwashing	508	-0.04 (1.02)	-0.07 (-0.17, 0.04)	143	-0.01 (1.04)	-0.03 (-0.24, 0.19)	0.756
WSH	549	-0.05 (0.98)	-0.03 (-0.14, 0.08)	120	0.11 (0.97)	0.06 (-0.14, 0.27)	0.477
Nutrition	512	0.00 (0.97)	-0.02 (-0.14, 0.11)	137	0.11 (0.93)	0.07 (-0.14, 0.28)	0.476
Nutrition + WSH	563	-0.01 (0.97)	0.00 (-0.09, 0.10)	148	0.12 (0.97)	0.07 (-0.13, 0.27)	0.565
N + WSH vs WSH			0.01 (-0.13, 0.15)			0.02 (-0.27, 0.31)	0.937
N + WSH vs N			0.00 (-0.13, 0.13)			0.02 (-0.22, 0.27)	0.879
**Combined z-score**							
Active Control	1146	-0.05 (1.01)		268	0.21 (0.91)		
Passive Control	530	-0.04 (0.98)	0.02 (-0.08, 0.11)	126	0.07 (1.00)	-0.07 (-0.29, 0.15)	0.505
Water	532	-0.09 (1.02)	-0.02 (-0.15, 0.10)	150	0.27 (0.87)	0.11 (-0.05, 0.27)	0.200
Sanitation	532	-0.15 (1.02)	-0.07 (-0.16, 0.03)	138	0.01 (0.98)	-0.07 (-0.25, 0.12)	0.989
Handwashing	508	-0.04 (0.99)	-0.04 (-0.14, 0.07)	143	-0.02 (1.12)	-0.13 (-0.35, 0.08)	0.428
WSH	549	-0.03 (0.96)	0.02 (-0.09, 0.13)	120	0.18 (0.88)	0.07 (-0.12, 0.25)	0.643
Nutrition	512	0.01 (1.01)	0.03 (-0.09, 0.16)	137	0.18 (0.84)	0.02 (-0.18, 0.22)	0.917
Nutrition + WSH	563	-0.07 (0.97)	-0.01 (-0.11, 0.09)	148	0.15 (0.97)	0.03 (-0.16, 0.21)	0.760
N + WSH vs WSH			-0.06 (-0.19, 0.07)			-0.03 (-0.28, 0.22)	0.841
N + WSH vs N			-0.06 (-0.19, 0.08)			-0.01 (-0.25, 0.23)	0.759

* p<0.05, ** p<0.01, *** p<0.001

Supplementary Table 12: CONSORT abstract checklist

Item	Standard Checklist item	Extension for cluster trials	Reported in section:
Title	Identification of study as randomised	Identification of study as cluster randomised	In Title
Trial design	Description of the trial design (e.g. parallel, cluster, non-inferiority)		In Methods
Methods			
Participants	Eligibility criteria for participants and the settings where the data were collected	Eligibility criteria for clusters	In Methods
Interventions	Interventions intended for each group		In Methods
Objective	Specific objective or hypothesis	Whether objective or hypothesis pertains to the cluster level, the individual participant level or both	In Background
Outcome	Clearly defined primary outcome for this report	Whether the primary outcome pertains to the cluster level, the individual participant level or both	In Methods
Randomization	How participants were allocated to interventions	How clusters were allocated to interventions	In Methods
Blinding (masking)	Whether or not participants, care givers, and those assessing the outcomes were blinded to group assignment		In Methods
Results			
Numbers randomized	Number of participants randomized to each group	Number of clusters randomized to each group	In Findings
Numbers analysed	Number of participants analysed in each group	Number of clusters analysed in each group	In Findings
Outcome	For the primary outcome, a result for each group and the estimated effect size and its precision	Results at the cluster or individual participant level as applicable for each primary outcome	In Findings
Harms	Harms or side effects		Not applicable
Conclusions	General interpretation of the results		In Interpretation
Trial registration	Registration number and name of trial register		In Methods
Funding	Source of funding		In Funding

Supplementary Table 13: CONSORT Checklist

Section/Topic	Item No	Standard Checklist item	Extension for cluster designs	Reported in section:
Title and abstract				
	1a	Identification as a randomised trial in the title	Identification as a cluster randomised trial in the title	Title
	1b	Structured summary of trial design, methods, results, and conclusions (for specific guidance see CONSORT for abstracts)	See table 2	Abstract
Introduction				
Background and objectives	2a	Scientific background and explanation of rationale	Rationale for using a cluster design	Introduction
	2b	Specific objectives or hypotheses	Whether objectives pertain to the the cluster level, the individual participant level or both	Introduction, last paragraph
Methods				
Trial design	3a	Description of trial design (such as parallel, factorial) including allocation ratio	Definition of cluster and description of how the design features apply to the clusters	Methods (Study design)
	3b	Important changes to methods after trial commencement (such as eligibility criteria), with reasons		Methods (Procedures, Last sentence of 3rd paragraph)
Participants	4a	Eligibility criteria for participants	Eligibility criteria for clusters	Methods (Participants)
	4b	Settings and locations where the data were collected		Methods (Study design)
Interventions	5	The interventions for each group with sufficient details to allow replication, including how and when they were actually administered	Whether interventions pertain to the cluster level, the individual participant level or both	Methods (Procedures)
Outcomes	6a	Completely defined pre-specified primary and secondary outcome measures, including how and when they were assessed	Whether outcome measures pertain to the cluster level, the individual participant level or both	Methods (Outcomes)
	6b	Any changes to trial outcomes after the trial commenced, with reasons		
Sample size	7a	How sample size was determined	Method of calculation, number of clusters(s) (and whether equal or unequal cluster sizes are assumed), cluster size, a coefficient of intracluster correlation (ICC or *k*), and an indication of its uncertainty	Methods (Statistical analysis, 1st paragraph)
	7b	When applicable, explanation of any interim analyses and stopping guidelines		Not applicable
Randomisation:				
Sequence generation	8a	Method used to generate the random allocation sequence		Methods (Randomization and masking)
	8b	Type of randomisation; details of any restriction (such as blocking and block size)	Details of stratification or matching if used	Methods (Randomization and masking)
Allocation concealment mechanism	9	Mechanism used to implement the random allocation sequence (such as sequentially numbered containers), describing any steps taken to conceal the sequence until interventions were assigned	Specification that allocation was based on clusters rather than individuals and whether allocation concealment (if any) was at the cluster level, the individual participant level or both	Methods (Randomization and masking)

Implementation	10	Who generated the random allocation sequence, who enrolled participants, and who assigned participants to interventions	Replace by 10a, 10b and 10c	
	10a		Who generated the random allocation sequence, who enrolled clusters, and who assigned clusters to interventions	Methods (Randomization and masking)
	10b		Mechanism by which individual participants were included in clusters for the purposes of the trial (such as complete enumeration, random sampling)	Methods (Participants)
	10c		From whom consent was sought (representatives of the cluster, or individual cluster members, or both), and whether consent was sought before or after randomisation	Methods (Study design, last paragraph)
Blinding	11a	If done, who was blinded after assignment to interventions (for example, participants, care providers, those assessing outcomes) and how		Methods (Randomization and masking)
	11b	If relevant, description of the similarity of interventions		Methods (Procedures)
Statistical methods	12a	Statistical methods used to compare groups for primary and secondary outcomes	How clustering was taken into account	Methods (Statistical analysis, 2nd and 3rd paragraphs)
	12b	Methods for additional analyses, such as subgroup analyses and adjusted analyses		Methods (Statistical analysis, 3rd paragraph)<br>Supplemental material
Results				
Participant flow (a diagram is strongly recommended)	13a	For each group, the numbers of participants who were randomly assigned, received intended treatment, and were analysed for the primary outcome	For each group, the numbers of clusters that were randomly assigned, received intended treatment, and were analysed for the primary outcome	Figure 1
	13b	For each group, losses and exclusions after randomisation, together with reasons	For each group, losses and exclusions for both clusters and individual cluster members	Figure 1
Recruitment	14a	Dates defining the periods of recruitment and follow-up		Results, 1st paragraph
	14b	Why the trial ended or was stopped		Not applicable
Baseline data	15	A table showing baseline demographic and clinical characteristics for each group	Baseline characteristics for the individual and cluster levels as applicable for each group	Table 1
Numbers analysed	16	For each group, number of participants (denominator) included in each analysis and whether the analysis was by original assigned groups	For each group, number of clusters included in each analysis	Figure 1
Outcomes and estimation	17a	For each primary and secondary outcome, results for each group, and the estimated effect size and its precision (such as 95% confidence interval)	Results at the individual or cluster level as applicable and a coefficient of intracluster correlation (ICC or k) for each primary outcome	Results 4th and 5th paragraphs<br>Tables 2-4<br>Figure 2
	17b	For binary outcomes, presentation of both absolute		Not applicable

		and relative effect sizes is recommended		
Ancillary analyses	18	Results of any other analyses performed, including subgroup analyses and adjusted analyses, distinguishing pre-specified from exploratory		Supplementary material
Harms	19	All important harms or unintended effects in each group (for specific guidance see CONSORT for harms)		Not applicable
Discussion				
Limitations	20	Trial limitations, addressing sources of potential bias, imprecision, and, if relevant, multiplicity of analyses		Discussion, 2nd to last paragraph
Generalisability	21	Generalisability (external validity, applicability) of the trial findings	Generalisability to clusters and/or individual participants (as relevant)	Discussion, last paragraph
Interpretation	22	Interpretation consistent with results, balancing benefits and harms, and considering other relevant evidence		Discussion section
Other information				
Registration	23	Registration number and name of trial registry		Methods (Statistical analysis, last paragraph)
Protocol	24	Where the full trial protocol can be accessed, if available		Reference 15<br>Methods (Study design, first sentence)
Funding	25	Sources of funding and other support (such as supply of drugs), role of funders		Methods (Role of the funding source)<br>Acknowledgements
